# Interaction among apoptosis-associated sequence variants and joint effects on aggressive prostate cancer

**DOI:** 10.1186/1755-8794-5-11

**Published:** 2012-04-30

**Authors:** Nicole A Lavender, Erica N Rogers, Susan Yeyeodu, James Rudd, Ting Hu, Jie Zhang, Guy N Brock, Kevin S Kimbro, Jason H Moore, David W Hein, La Creis R Kidd

**Affiliations:** 1 Department of Pharmacology & Toxicology, School of Medicine, University of Louisville (UofL), Louisville, KY, USA; 2 Department of Biology, The Julius L. Chambers Biomedical/Biotechnology Research Institute, North Carolina Central University, Durham, NC, USA; 3 Departments of Genetics and Community and Family Medicine, Institute for Quantitative Biomedical Sciences, Norris Cotton Cancer Center, Dartmouth Medical School, Lebanon, NH, USA; 4 Department of Bioinformatics & Biostatistics, School of Public Health and Information Sciences, UofL, Louisville, KY, USA; 5 505 South Hancock Street, Clinical & Translational Research Building, Room 306, Louisville, KY 40202, USA

**Keywords:** Prostate cancer, Apoptosis, Single nucleotide polymorphisms, Gene-gene interactions, Multifactor dimensionality reduction (MDR), Statistical epistasis networks (SEN)

## Abstract

**Background:**

Molecular and epidemiological evidence demonstrate that altered gene expression and single nucleotide polymorphisms in the apoptotic pathway are linked to many cancers. Yet, few studies emphasize the interaction of variant apoptotic genes and their joint modifying effects on prostate cancer (PCA) outcomes. An exhaustive assessment of all the possible two-, three- and four-way gene-gene interactions is computationally burdensome. This statistical conundrum stems from the prohibitive amount of data needed to account for multiple hypothesis testing.

**Methods:**

To address this issue, we systematically prioritized and evaluated individual effects and complex interactions among 172 apoptotic SNPs in relation to PCA risk and aggressive disease (i.e., Gleason score ≥ 7 and tumor stages III/IV). Single and joint modifying effects on PCA outcomes among European-American men were analyzed using statistical epistasis networks coupled with multi-factor dimensionality reduction (SEN-guided MDR). The case-control study design included 1,175 incident PCA cases and 1,111 controls from the prostate, lung, colo-rectal, and ovarian (PLCO) cancer screening trial. Moreover, a subset analysis of PCA cases consisted of 688 aggressive and 488 non-aggressive PCA cases. SNP profiles were obtained using the NCI Cancer Genetic Markers of Susceptibility (CGEMS) data portal. Main effects were assessed using logistic regression (LR) models. Prior to modeling interactions, SEN was used to pre-process our genetic data. SEN used network science to reduce our analysis from > 36 million to < 13,000 SNP interactions. Interactions were visualized, evaluated, and validated using entropy-based MDR. All parametric and non-parametric models were adjusted for age, family history of PCA, and multiple hypothesis testing.

**Results:**

Following LR modeling, eleven and thirteen sequence variants were associated with PCA risk and aggressive disease, respectively. However, none of these markers remained significant after we adjusted for multiple comparisons. Nevertheless, we detected a modest synergistic interaction between *AKT3 rs2125230-PRKCQ rs571715 *and disease aggressiveness using SEN-guided MDR (p = 0.011).

**Conclusions:**

In summary, entropy-based SEN-guided MDR facilitated the logical prioritization and evaluation of apoptotic SNPs in relation to aggressive PCA. The suggestive interaction between *AKT3-PRKCQ *and aggressive PCA requires further validation using independent observational studies.

## Background

Prostate cancer (PCA) is the most frequently diagnosed cancer and the 2^nd ^leading cause of cancer-related deaths among men in the United States [1]. The American Cancer Society estimates that 26-29% of all new cancer cases and cancer-related deaths are attributed to PCA cancer. Well established PCA risk factors include older age, black race, and family history of PCA. However, other potential contributors of this disease may include lifestyle and genetic factors as well as imbalances within important biological pathways.

Apoptosis or programmed cell death is one such biological process that moderates cell differentiation, proliferation, death, whole body homeostasis and tumorigenesis [2-4]. This process is controlled by cell death (e.g. BAD, CASP, BIK) and cell survival proteins (e.g. BCL2, NFκB, AKT3) that induce or block apoptosis, respectively, as summarized in Table 1[2,3,5]. Decreased apoptotic cell death and increased cell proliferation may lead to clonal expansion and tumor growth [2]. Failure to undergo apoptosis permits survival of transformed cells, leading to subsequent genetic alteration, genomic instability, and ultimately a more invasive cancer phenotype [3]. Imbalances in apoptosis-related genes may play an important role in PCA susceptibility as well as disease progression. For example, several independent studies have shown that overexpression of cell survival indicators (e.g., BCL-2, CARD8, IKBKE, PRKCQ, and PIK3CB, AKT3) or down-regulation of cell death markers (e.g., BCL2L14) are associated with more aggressive phenotypes, higher Gleason grade, increased tumor progression, and poor PCA prognosis [6-19].

**Table 1:** Selected genes involved in the regulation of apoptosis

	Gene	Function*
Pro- & Anti- apoptotic	*Tumor Protein 53 (TP53)*	Transcriptionally regulates target genes that induce cell cycle arrest, apoptosis, senescence, DNA repair, or changes in metabolism in response to cellular stresses.
	
	*Tumor Necrosis Factor* *(TNF)*	Binds and functions through its receptors *TNFRSF1A/TNFR1 *and *TNFRSF1B/TNFBR *to regulate cell proliferation, differentiation, and apoptosis.
	
	*PRKCQ*	Protein kinase C family member; substrate for Caspase-3; phosphorylates BAD and required to activate NFκB (via CARD11 phosphorylation) and AP-1.

Pro-apoptotic	*FAS *&*FAS Ligand (FASL)*	Death domain-containing receptor, binding of FASL to FAS allows the formation of a death-inducing signaling complex.
	
	*CASPASE (CASP)*	Gene family involved in the execution of apoptosis. There are 2 classes of caspases, which include: initiators (e.g., *CASP2, CASP8, CASP9, and CASP10*) and effectors (e.g., *CASP3, CASP6, CASP7*). Initiator caspases activate pro-forms of effector caspases, enabling effectors to trigger the apoptosis process.
	
	*CARD8 (Caspase* *recruitment domain* *family, member 8)*	CARD family protein; involved in various pathways which regulate caspases or NFκB; isoforms interact with caspases to signal apoptosis.
	
	*BCL2-associated X (BAX)*	Forms a heterodimer with *BCL2 *and functions as an apoptotic activator involved mitochondrial release of cytochrome c.
	
	*BCL2-antagonist/killer 1* *(BAK1)*	Induces apoptosis by increasing cytochrome c release; interacts with the *TP53 *after exposure to cell stress.
	
	*BCL2-associated agonist of* *cell death (BAD)*	Forms heterodimers with *BCLXL *and *BCL2 *to reverse their death repressor activity.
	
	*BCL2-like 10 (BCL2L10)*	Interacts with *BCL2 *proteins (e.g., *BCL2, BCL2L1/BCLXL*, and *BAX*).
	
	*BCL2-like 11 (BCL2L11)*	(aka *BIM*); Interacts with other members of the *BCL2 *protein family (e.g., *BCL2, BCL2L1/BCLXL*, and *MCL1*) to act as an apoptosis activator.
	
	*BCL2-like 14 (BCL2L14)*	Apoptosis facilitator; interacts with BCL2 family members; p53- target gene.
	
	*BH3 interacting domain* *death agonist (BID)*	Induced by *CASP8*; *CASP8 *cleaves the protein encoded by this gene, and the COOH-terminal part translocates to mitochondria, which triggers cytochrome c release.
	
	*BCL2-interacting killer* *(BIK)*	Interacts with survival-promoting proteins to enhance programmed cell death.
	
	*BCL2/adenovirus E1B* *19 kDa interacting protein* *3-like (BNIP3L)*	(aka *NIX*); *BCL2/adenovirus E1B 19 kd-interacting protein (BNIP)* gene that may function simultaneously with *BNIP3 *and play a role in tumor suppression.
	
	*PRKCD*	Translocates into nucleus during apoptosis. Nuclear PRKCD regulates initiation of cytosolic apoptosis machinery, and subsequent caspase activation and DNA fragmentation.

Anti-apoptotic	*AKT3*	Phosphorylate and inactivate BAD. Activates NFκB via IκB kinase regulation. Regulates cell signals in response to insulin and growth factors.
	
	*B-cell CL/lymphoma 2 (BCL2)*	Blocks the release of pro-apoptotic cytochrome c and caspase activation.
	
	NFκB	Inhibit caspases 3, 6, 7 stimulation via IAP (inhibitor of apoptosis) activation.
	
	*PIK3CB*	Interacts with growth factor receptors; activates AKT3; target for PRKC.
	
	*RAF1*	Inhibits BIM and BAD activation via ERK1/2 stimulation.
	
	*BCL2-related protein A1 (BCL2A1)*	Reduces cytochrome c release from mitochondria and blocks caspase activation.
	
	*Baculoviral IAP repeat-containing 2 (BIRC2)*	(aka *CIAP1*); Inhibits apoptosis by binding to tumor necrosis factor receptor-associated factors *TRAF1 *and *TRAF2*.
	
	*PRKCE*	Blocks mitochondrial-dependent caspase activation; phosphorylates and activates RAF-1; phosphorylates and inactivates BAD; activates AKT via DNA-dependent protein kinase (DNA-PK).
	
	*IKBKE*	Induces BCL-2 expression via NFκB signaling and interaction.

*Gene functions based on NCBI and/or selected publications (as indicated in the manuscript)

There is mounting epidemiological evidence that genetic alterations in apoptosis-related genes play an important role in tumorigenesis. Apoptosis-associated sequence variants, when considered individually, may minimally influence the risk of developing numerous cancers, such as multiple myeloma, squamous cell carcinoma, chronic lymphocytic leukemia, non-Hodgkin lymphoma, colorectal, ovarian, breast, pancreatic, and non-small cell lung [20-37]. However, the impact of individual apoptosis-related single nucleotide polymorphisms (SNPs) and their interactions on PCA outcomes remains understudied.

Genome wide association studies (GWAS), involving the evaluation of millions of SNPs within various biological pathways, has resulted in the detection of numerous PCA susceptibility loci [38]. However, most GWAS and PCA epidemiology studies place emphasis on individual SNP effects. Consequently, researchers often ignore the fact that complex diseases such as PCA are governed by complex gene-gene and gene-environment interactions within distinct biological pathways. Consequently, we sought to evaluate millions of interactions among apoptosis SNPs and their joint modifying impact on PCA risk and disease progression.

This report focuses on the impact of complex interactions among 172 apoptosis-related SNPs on PCA among 2,286 European-American male participants of the Cancer Genetic Markers of Susceptibility Study (CGEMS). The exhaustive assessment of all possible two-, three-, and four-way interactions among 172 SNPs, totaling more than 36 million SNP combinations is computationally burdensome. The statistical challenge stems from the prohibitive amount of data needed for multiple hypothesis testing. To address this issue, for the first time, we use statistical epistasis networks (SEN) guided multi-factor dimensionality reduction (MDR) to efficiently pre-process our genetic data, prior to modeling higher order interactions. As previously reported by Hu and co-workers (2011), SEN uses network science to generate a large connected cluster of pairwise interactions embedded within a genetic dataset [39]. The resulting network, consisting of a subset of high susceptibility SNPs, is used to guide SNP interactions using MDR. MDR is a rigorous statistical tool designed, in part, to evaluate main effects and complex interactions in relation to discrete outcomes [40-48]. This report may serve as a foundation for researchers interested in a state-of the art bioinformatics technique, namely SEN-guided MDR, which facilitates the logical prioritization of SNPs for gene-gene interaction analyses in relation to PCA susceptibility and prognosis.

## Results

### Cancer Genetic Markers of Susceptibility (CGEMS) Population Description

CGEMS study participants consisted of middle aged non-Hispanic Caucasian men, ranging in age from 55 to 81. Relative to control subjects, men diagnosed with PCA were more likely to have a family history of the disease (11.4% versus 6.3%), PSA levels ≥ 4 ng/ml (48.5% versus 6.5%), and abnormal DRE exams (60.7% versus 46.2%) [data not shown]. Men with aggressive PCA had both a Gleason score ≥ 7 and tumor stage III/IV. Although non-aggressive PCA cases primarily had a Gleason score < 7 and tumor stage I/II, a small percentage (1.8%) of them had a Gleason score > 6 and a tumor stage I/II. Among cases, there were no significant differences in family history of disease or PSA levels between men diagnosed comparing men with aggressive and non-aggressive disease (data not shown). The vast majority of subjects with aggressive PCA had higher Gleason scores than subjects with non-aggressive PCA, although 9 of the 488 non-aggressive cases (1.08%) had a Gleason score greater than 6.

### Single SNP Effects

We examined 172 apoptosis-related sequence variants in relation to PCA risk and disease aggressiveness, as shown in Tables 2 and 3. Age and family history of PCA did not modify the relationship between the sequence variants and the risk of developing PCA or aggressive disease. Inheritance of one or more *TNFRSF10B rs1001793 *or *TNFSF10 rs4894559 *A alleles was nominally associated with a 1.23-1.38 fold increase in PCA risk (p ≤ 0.039), as shown in Table 2. Whereas, eight apoptosis-related SNPs were associated with a moderate 18-50% reduction in disease susceptibility [*BIK rs4988366 AG & AG + GG*; *BNIP3L rs10503786 TT & TC + TT; CASP9 rs1052571 TT*; *IKBKE rs1539243 TC & TC + TT; PRKCE rs608139 CC; PRKCE rs935673 GG & AG + GG; *TNFRSF1A *rs4149576 AG, GG, & AG + GG*; and *TNFRSF1A rs4149577 TC & TC + CC*] (p ≤ 0.043). However, after applying Bonferonni correction, none of these findings remained statistically significant under the per-allele, dominant and recessive genetic models.

**Table 2:** Association of apoptosis SNPs with prostate cancer

Marker (Alleles and position)	Allele	Cases N (%)	Controls N (%)	OR (95%CI)	Adj OR (95%CI)*	p value	**Bonf**. p value	p trend	**Permut**. p value
*BIK*	AA	944 (81.2)	855 (77.0)	1.00 (reference)	1.00 (reference)	0.048	1.000	0.017	1.000

rs4988366	AG	208 (17.9)	244 (22.0)	0.77 (0.63-0.95)	0.78 (0.63-0.96)	0.014			

A41830193G	GG	12 (1.0)	14 (1.3)	0.78 (0.36-1.69)	0.79 (0.36-1.71)	0.523			

	AG + GG	220 (18.9)	258 (23.2)	0.78 (0.63-0.95)	0.78 (0.64-0.96)	0.014	1.000		0.831

*BNIP3L*	CC	556 (47.8)	477 (42.9)	1.00 (reference)	1.00 (reference)	0.041	1.000	0.012	0.998

rs10503786	TC	480 (41.3)	489 (44.0)	0.84 (0.71-1.00)	0.85 (0.71-1.01)	0.055			

C26325853T	TT	103 (8.9)	122 (11.0)	0.72 (0.54-0.97)	0.73 (0.55-0.98)	0.029			

	TC + TT	583 (50.1)	611 (55.0)	0.82 (0.70-0.97)	0.82 (0.70-0.97)	0.020	1.000		0.928

*CASP9*	CC	320 (27.5)	348 (31.3)	1.00 (reference)	1.00 (reference)	0.075	1.000	0.023	1.000

rs1052571	TC	589 (50.6)	553 (49.8)	0.88 (0.71-1.10)	0.88 (0.71-1.09)	0.265			

C15595919T	TT	253 (21.8)	210 (18.9)	0.76 (0.60-0.97)	0.75 (0.59-0.95)	0.026			

	TC + CC	842 (72.4)	763 (68.7)	0.84 (0.68-1.03)	0.83 (0.68-1.02)	0.090	1.000		0.998

*IKBKE*	CC	837 (72.0)	740 (66.6)	1.00 (reference)	1.00 (reference)	0.017	1.000	0.005	0.903

rs1539243	TC	291 (25.0)	325 (29.3)	0.79 (0.66-0.95)	0.78 (0.65-0.95)	0.014			

C203036182T	TT	36 (3.1)	47 (4.2)	0.68 (0.43-1.06)	0.68 (0.43-1.06)	0.086			

	TC + TT	327 (28.1)	372 (33.5)	0.78 (0.65-0.93)	0.77 (0.64-0.92)	0.006	0.943		0.522

*PRKCE*	TT	841 (72.3)	788 (70.9)	1.00 (reference)	1.00 (reference)	0.030	1.000	0.139	0.987

rs608139	TC	304 (26.1)	288 (25.9)	0.99 (0.82-1.19)	0.98 (0.51-1.19)	0.909			

T45789207C	CC	18 (1.5)	36 (3.2)	0.47 (0.26-0.83)	0.50 (0.28-0.88)	0.010			

	TC + CC	322 (27.7)	324 (29.2)	0.93 (0.78-1.12)	0.93 (0.78-1.12)	0.435	1.000		1.000

*PRKCE*	AA	556 (47.8)	482 (43.4)	1.00 (reference)	1.00 (reference)	0.045	1.000	0.013	1.000

rs935673	AG	503 (43.3)	503 (45.3)	0.87 (0.73-1.03)	0.87 (0.73-1.04)	0.107			

A46034008G	GG	105 (9.0)	128 (11.5)	0.71 (0.53-0.95)	0.71 (0.53-0.95)	0.019			

	AG + GG	608 (52.3)	631 (56.8)	0.84 (0.71-0.99)	0.84 (0.71-0.99)	0.034	1.000		0.991

*TNFRSF10B*	GG	547 (47.0)	537 (48.3)	1.00 (reference)	1.00 (reference)	0.055	1.000	0.253	1.000

rs1001793	AG	448 (38.5)	458 (41.2)	0.96 (0.80-1.15)	0.96 (0.80-1.14)	0.653			

G22956894A	AA	131 (11.3)	93 (8.4)	1.38 (1.03-1.85)	1.38 (1.03-1.85)	0.029			

	AG + AA	579 (49.8)	551 (49.6)	1.01 (0.86-1.19)	1.03 (0.87-1.22)	0.892	1.000		1.000

*TNFRSF1A*	AA	375 (32.2)	376 (33.8)	1.00 (reference)	1.00 (reference)	0.079	1.000	0.070	1.000

rs4149576	AG	545 (46.9)	545 (79.1)	0.78 (0.62-0.98)	0.79 (0.63-0.99)	0.034			

A6319376G	GG	234 (20.1)	183 (16.5)	0.78 (0.61-0.99)	0.79 (0.62-1.01)	0.043			

	AG + GG	779 (67.0)	728 (65.5)	0.78 (0.63-0.97)	0.79 (0.63-0.98)	0.024	1.000		0.854

*TNFRSF1A*	TT	326 (28.0)	270 (24.3)	1.00 (reference)	1.00 (reference)	0.087	1.000	0.205	1.000

rs4149577	TC	571 (49.1)	591 (5302)	0.80 (0.66-0.98)	0.80 (0.66-0.98)	0.027			

T6317783C	CC	265 (22.8)	251 (22.6)	0.87 (0.69-1.11)	0.88 (0.70-1.12)	0.266			

	TC + CC	836 (71.9)	842 (75.8)	0.82 (0.68-0.99)	0.83 (0.68-1.00)	0.038	1.000		0.141

*TNFSF10*	GG	630 (54.2)	668 (60.1)	1.00 (reference)	1.00 (reference)	0.026	1.000	0.014	0.977

rs4894559	AG	456 (39.2)	380 (34.2)	1.27 (1.07-1.51)	1.27 (1.06-1.51)	0.007			

G173716071A	AA	76 (6.5)	65 (5.9)	1.24 (0.88-1.78)	1.23 (0.87-1.74)	0.227			

	AG + AA	532 (45.7)	445 (40.1)	1.26 (1.06-1.49)	1.26 (1.07-1.49)	0.007	1.000		0.594

Abbreviations: Bonf., Bonferroni; Permut., Permutation; *adjusted for age and family history of PCA

**Table 3:** Association of apoptosis SNPs with aggressive prostate cancer

Marker (Alleles and position)	Allele	Cases N (%)	Controls N (%)	OR (95%CI)	Adj OR (95%CI)*	p value	**Bonf**. p value	p trend	**Permut**. p value
*AKT3*	CC	295 (42.9)	244 (50.0)	1.00 (reference)	1.00 (reference)	0.065	1.000	0.028	1.000

rs10157763	TC	307 (44.7)	192 (39.3)	1.31 (1.02-1.67)	1.33 (1.04-1.71)	0.027			

C240321082T	TT	75 (10.9)	45 (9.2)	1.21 (0.82-1.78)	1.39 (0.92-2.09)	0.344			

	TC + TT	382 (55.6)	237 (48.6)	1.32 (1.04-1.66)	1.34 (1.06-1.70)	0.020	1.000		0.943

*AKT3*	GG	438 (63.8)	345 (70.7)	1.00 (reference)	1.00 (reference)	0.005	0.853	0.038	0.493

rs10927067	AG	227 (33.0)	119 (24.4)	1.50 (1.15-1.95)	1.51 (1.16-1.96)	0.002			

G240249285A	AA	20 (2.9)	19 (3.9)	0.83 (0.44-1.58)	0.82 (0.43-1.57)	0.568			

	AG + AA	247 (36.0)	138 (28.3)	1.43 (1.12-1.84)	1.41 (1.10-1.82)	0.005	0.977		0.511

*AKT3*	TT	483 (70.3)	380 (77.9)	1.00 (reference)	1.00 (reference)	0.002	0.356	0.023	0.250

rs12031994	TC	192 (27.9)	94 (19.3)	1.61 (1.21-2.13)	1.61 (1.22-2.14)	0.009			

T240243350C	CC	13 (1.9)	14 (2.9)	0.73 (0.34-1.57)	0.73 (0.34-1.57)	0.422			

	TC + CC	205 (29.8)	108 (22.1)	1.51 (1.15-1.98)	1.50 (0.15-1.96)	0.003	0.548		0.372

*AKT3*	GG	436 (63.5)	341 (69.9)	1.00 (reference)	1.00 (reference)	0.030	1.000	0.068	0.984

rs2125230	AG	231 (33.6)	129 (26.4)	1.40 (1.09-1.81)	1.40 (1.08-1.82)	0.010			

G240211889A	AA	21 (3.1)	18 (3.7)	0.91 (0.48-1.74)	0.91 (0.48-1.74)	0.781			

	AG + AA	252 (36.7)	147 (30.1)	1.35 (1.06-1.73)	1.34 (1.05-1.72)	0.017	1.000		0.938

*AKT3*	GG	296 (43.1)	238 (48.8)	1.00 (reference)	1.00 (reference)	0.083	1.000	0.027	1.000

rs2125231	AG	314 (45.7)	203 (41.6)	1.24 (0.97-1.59)	1.25 (0.98-1.60)	0.082			

G240088340A	AA	75 (10.9)	42 (8.6)	1.43 (0.95-2.17)	1.44 (0.95-2.18)	0.087			

	AG + AA	389 (56.6)	245 (50.2)	1.29 (1.02-1.63)	1.28 (1.01-1.62)	0.031	1.000		0.992

*AKT3*	CC	295 (42.9)	243 (49.8)	1.00 (reference)	1.00 (reference)	0.060	1.000	0.022	1.000

rs2345994	TC	312 (45.4)	195 (40.0)	1.32 (1.03-1.69)	1.32 (1.03-1.70)	0.028			

C240258316T	TT	78 (11.4)	45 (9.2)	1.43 (0.95-2.14)	1.44 (0.95-2.16)	0.084			

	TC + TT	390 (56.8)	240 (49.2)	1.32 (1.05-1.67)	1.35 (1.06-1.70)	0.018	1.000		0.946

*AKT3*	CC	435 (63.3)	348 (71.3)	1.00 (reference)	1.00 (reference)	0.009	1.000	0.029	0.714

rs4132509	AC	227 (33.0)	121 (24.8)	1.50 (1.15-1.95)	1.51 (1.16-1.96)	0.002			

C240269125A	AA	18 (2.6)	16 (3.3)	0.90 (0.45-1.79)	0.88 (0.44-1.75)	0.764			

	AC + AA	245 (35.7)	137 (28.1)	1.43 (1.11-1.84)	1.44 (1.11-1.85)	0.005	1.000		0.581

*BCL2L14*	GG	436 (63.5)	343 (70.3)	1.00 (reference)	1.00 (reference)	0.041	1.000	0.025	0.998

rs885720	AG	231 (33.6)	131 (26.8)	1.39 (1.07-1.79)	1.39 (1.08-1.80)	0.012			

G12139366A	AA	21 (3.1)	14 (2.9)	1.18 (0.59-2.25)	1.20 (0.60-2.39)	0.639			

	AG + AA	252 (36.7)	145 (29.7)	1.37 (1.07-1.76)	1.37 (1.07-1.76)	0.013	1.000		0.843

*CARD8*	CC	462 (67.2)	362 (74.2)	1.00 (reference)	1.00 (reference)	0.016	1.000	0.005	0.882

rs10405717	TC	202 (29.4)	111 (22.7)	1.45 (1.11-1.90)	1.45 (1.11-1.90)	0.007			

C53443620T	TT	21 (3.1)	10 (2.0)	1.70 (0.79-3.68)	1.70 (0.79-3.68)	0.173			

	TC + TT	223 (32.5)	121 (24.8)	1.45 (1.12-1.89)	1.47 (1.13-1.91)	0.005	0.754		0.459

*IKBKE*	TT	251 (36.5)	209 (42.8)	1.00 (reference)	1.00 (reference)	0.008	1.000	0.003	0.682

rs11578093	TG	332 (48.3)	233 (47.7)	1.19 (0.93-1.52)	1.17 (0.91-1.50)	0.177			

T203059049G	GG	102 (6.0)	46 (9.4)	1.85 (1.25-2.74)	1.81 (1.22-2.69)	0.002			

	TG + GG	434 (63.2)	279 (57.2)	1.31 (1.03-1.66)	1.27 (1.00-1.62)	0.027	1.000		0.982

*IKBKE*	CC	502 (73.1)	341 (69.9)	1.00 (reference)	1.00 (reference)	0.048	1.000	0.076	1.000

rs1539243	TC	172 (25.0)	125 (25.6)	0.93 (0.71-1.22)	0.95 (0.73-1.24)	0.622			

C203036182T	TT	14 (2.0)	22 (4.5)	0.43 (0.22-0.86)	0.44 (0.22-0.88)	0.016			

	TC + TT	186 (27.1)	147 (30.1)	0.86 (0.66-1.11)	0.88 (0.68-1.13)	0.244	1.000		1.000

*PIK3CB*	TT	215 (31.3)	131 (26.8)	1.00 (reference)	1.00 (reference)	0.060	1.000	0.021	1.000

rs500687	TC	334 (48.6)	234 (48.0)	0.87 (0.66-1.14)	0.87 (0.66-1.14)	0.318			

T139942901C	CC	134 (19.5)	121 (24.8)	0.67 (0.49-0.94)	0.66 (0.48-0.92)	0.019			

	TC + CC	468 (68.1)	355 (72.7)	0.81 (0.62-1.04)	0.80 (0.62-1.03)	0.099	1.000		1.000

*RAF1*	TT	508 (73.9)	377 (77.3)	1.00 (reference)	1.00 (reference)	0.010	1.000	0.473	0.764

rs13060691	TG	167 (24.3)	90 (18.4)	1.38 (1.03-1.84)	1.38 (1.03-1.84)	0.030			

T12653013G	GG	10 (1.5)	16 (3.3)	0.44 (0.20-0.97)	0.45 (0.20-1.01)	0.060			

	TG + GG	177 (25.8)	106 (21.7)	1.24 (0.94-1.63)	1.24 (0.94-1.63)	0.123	1.000		1.000

Abbreviations: Bonf., Bonferroni correction; Permut., Permutation; *adjusted for age and family history of PCA (PCA)

In terms of disease progression, eleven apoptosis SNPs were modestly associated with a 1.28-1.81 fold increase in the risk of developing aggressive PCA [*AKT3 rs10157763 TC & TC + TT; AKT3 rs10927067 AG & AG + AA; AKT3 rs12031994 TC & TC + CC;AKT3 rs2125230 AG & AG + AA; AKT3 rs2125231 AG + AA; AKT3 rs2345994 TC & TC + CC; AKT3 rs4132509 AC & AC + AA; BCL2L14 rs885720 AG & AG + AA; CARD8 rs10405717 TC & TC + TT; IKBKE rs11578093 GG & TG + GG; *and *RAF1 rs13060691 TG*] (p ≤ 0.031), as summarized in Table 3. In addition, possession of *IKBKE rs1539243 TT *and *PIK3CB rs500687 CC *genotypes were moderately linked with a 44-56% reduction in aggressive PCA susceptibility (p ≤ 0.019). However, none of these loci remained significant after adjusting for multiple comparison bias.

### Combined Gene Effects

**Figure 1:**
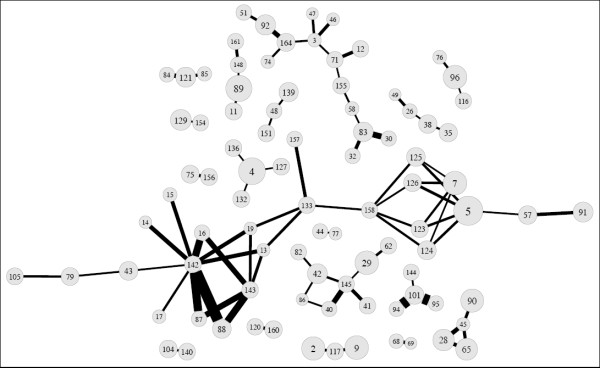
**Visualization of the interaction among apoptosis-related sequence variants using Statistical Epistasis Network Modeling (SEN)-guided MDR**. The Statistical Epistasis Network (SEN) was generated using a pairwise interaction strength cut-off or 1%. Overall, the model has 91 vertices (single SNPs), 80 edges (pairwise SNPs), and 18 components ("sub-networks" of vertices and edges). The largest connected component, shown in the center and spanning the entire length of this model, has 24 vertices and 34 edges. Each number in Figure 1 corresponds with a specific apoptosis-related SNP analyzed in the current study, as summarized in Additional file 2.

**Figure 2:**
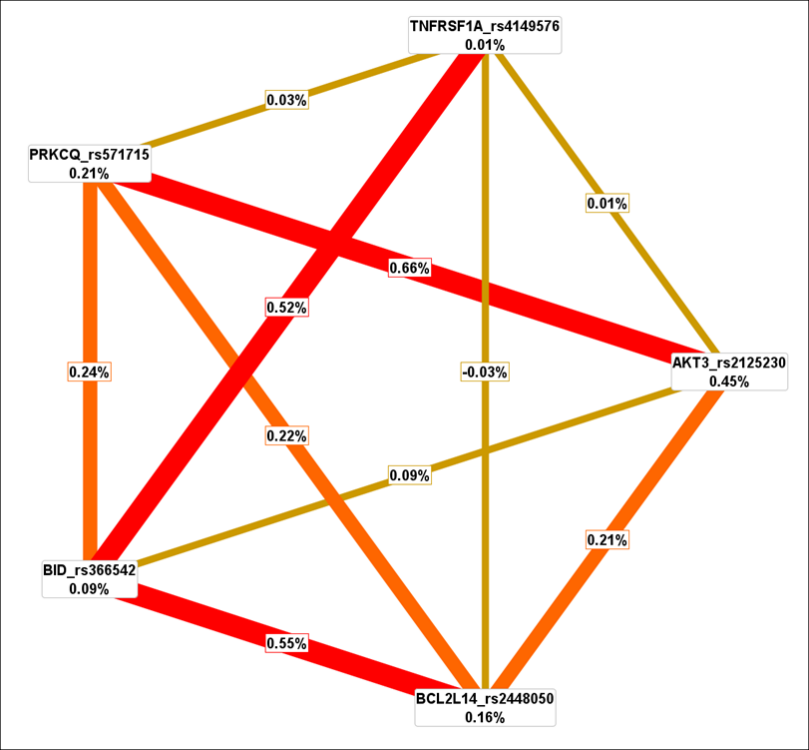
**Interaction Entropy model**. This graphical model, describes the percent entropy that is explained by each apoptosis-related SNP or pairwise combination within our study population. Positive percent entropy indicates information gain (IG) or synergy; whereas, negative percent indicates redundancy or lack of information gain (IG). Schematic coloration used in the visualization tools represents a continuum from synergy (i.e. non-additive) to redundancy. The colors range from red representing a high degree of synergy (positive information gain (IG)), orange a lesser degree, and gold representing independence and a midway point between synergy and redundancy. On the other hand, green and blue represent redundancy, which is not apparent in the current analysis.

We investigated the ability of gene-gene interactions to predict PCA outcomes using statistical epistasis networks (SEN) guided multifactor dimensionality reduction (MDR), information gain (IG) measure, and hierarchical interactions graphs (hIG). SEN was used to generate a network to visualize the genetic architecture of PCA outcomes, prior to an exhaustive search of SNP interactions. This approach uses information theory to build an epistasis network from the strongest pairwise SNP interactions. A network, consisting of vertices (i.e., SNPs) joined by edges (i.e., SNP pairs), enabled us to limit our MDR analysis to a subset of the most informative SNPs in relation to PCA outcomes. We were unable to build a statistically significant epistasis network for PCA susceptibility. However, the network for aggressive PCA was topographically significant (p < 0.05), given the results from 1000 permutated data sets. This network consisted of 91 vertices (i.e., individual SNP effects), 80 edges (i.e., pairwise interactions), and 18 total components (i.e., "sub-networks" of vertices and edges). The largest connected component of the aggressive PCA SEN diagram involved 24 vertices and 34 edges, as shown in Figure 1 andAdditional file 1. Consequently, MDR analysis was limited to < 13,000 interactions among 24 SNPs, instead of more than 36 million interactions for 172 SNPs. SEN-guided MDR revealed complex interactions between *AKT3 rs12031994-PRKCQ rs571715 *as well as *AKT3 rs12031994-BID rs366542-PRKCQ rs571715 *that were significantly associated with disease aggressiveness (p ≤ 0.009). However, the network and entropy graphs indicated these findings were mainly driven by *AKT3 rs12031994*. This is evident by the marker's large vertex size (i.e., large main effect) in the SEN graph (Figure 1) and a lack of a strong pairwise interaction between *AKT3 rs12031994-PRKCQ rs571715 *in the entropy graph. The *AKT3-PRKCQ *interaction resulted in joint mutual information (I) of only 0.54% relative to the mutual information (I) of 0.87% for *AKT3 *alone. Therefore, prior to repeating MDR, we removed the *AKT3rs12031994 *marker. This resulted in the detection of a modest synergistic interaction between *AKT3 rs2125230-PRKCQ rs571715 *(p = 0.011), as shown in Table 4. Based on MDR, this model was the best two-factor model. It was selected 9 out of 10 times by MDR based on a cross-validation consistency (CVC) score of 90% and predictive accuracy of 56.3%. Furthermore, we observed a higher mutual information for the combined effect of *AKT3 rs2125230-PRKCQ rs571715 *(I = 0.66%), in contrast to *AKT3 rs2125230 *alone (I = 0.45%) or *PRKCQrs571715 alone *(I = 0.21%), as revealed in Figure 2.

**Table 4:** Multi-dimensionality reduction models for apoptosis-related markers in relation to aggressive prostate cancer (PCA)

Best Model	Cross Validation Consistency (CVC)*	Average Testing Accuracy (ATA)*	Permutation Testing p value*
One Factor *AKT3 rs2125230*	9/10	0.512	0.282

Two Factor *AKT3 rs2125230* *PRKCQ rs571715*	9/10	0.563	0.011

Three Factor *AKT3 rs2125230* *BID rs366542* *PRKCQ rs571715*	6/10	0.529	0.127

Four Factor *AKT3 rs2125230* *BCL2L14 rs2448050* *BID rs366542* *TNFRSF1A rs4149576*	2/10	0.517	0.321

*Adjusted for age and family history of prostate cancer (PCA)

To confirm the validity of the SEN results, MDR analysis was also conducted on all 172 SNPs (Figure 1 andAdditional file 1) as well as 148 loci that were not included in the main component of the network. As expected, we did not detect any significant two-, three-, or four- way interaction models (p ≥ 0.184).

## Discussion

Previous genome-wide association studies (GWAS) have identified linkages about 40 PCA loci, including between PCA genetic alterations detected in the 8q24 region, β-microseminoprotein (*MSMB*), and allele -8 of the microsatellite DG8S737 [38]. These studies have limited their scope to individual SNPs across the entire genome. Such assessments tend to ignore the genetic architecture of PCA that potentially involves complex interactions along key regulatory pathways. For the first time, the current study evaluates complex interactions among 172 apoptosis-related SNPs in relation to PCA risk and disease aggressiveness among 2,286 European-American men using SEN-guided MDR. Specifically, SEN was used to build a topographically significant aggressive PCA epistasis network, prior to evaluating complex interactions. This inferred epistasis network consisted of 24 SNPs and 34 pairwise interactions, and reduced MDR analysis from > 36 million to < 13,000 SNP interactions. Consequently, we observed a non-linear and modest interaction between *AKT3 rs2125230-PRKCQ rs571715 *in relation to aggressive PCA. This state-of-the-art bioinformatics technique facilitates the logical prioritization of SNPs for gene-gene interaction analyses in relation to complex diseases.

Unfortunately, there are no published reports on the functional consequence of these two intronic SNPs in *AKT3 *and *PRKCQ *in relation to mRNA stability/expression protein expression/structure/function or PCA outcomes. However, we speculate that the *AKT3 rs2125230 *and *PRKCQ rs571715 *sequence variants, with minor allele frequencies ranging from 14.4-22.9 among men of European descent, may alter transcription regulation, leading to increased mRNA expression. Increased mRNA/protein expression *AKT3 rs2125230 *and *PRKCQ rs571715 *may cause: decreased apoptosis, an escape of transformed cells from programmed cell death, increased accumulation of genetic alterations, genomic instability, and ultimately an invasive PCA phenotype. Thus, *in vitro *and *in vivo *assays using (short hairpin RNAs) shRNAs or small interfering RNAs (siRNAs) are needed to elucidate the impact of *AKT3*-*PRKCQ *genetic alterations on protein expression, apoptosis capacity, and prostate tumorigenesis.

The impact of a non-linear interaction along the *AKT3 rs2125230-PRKCQ rs571715 *axis in relation to aggressive PCA may be attributed to markers involved in the apoptosis signaling pathway. Overexpression of PRKCQ and AKT3 are associated with invasive cancer phenotypes [6-19]. In fact, AKT3 is responds to insulin and growth factors, transduces signals including cell death, and is upregulated in androgen-independent PCA cell lines [49]. PRKCQ is also associated with apoptosis. In particular PRKCQ, a protein kinase C (PRKC) family member, promotes cell survival by inactivating BAD (BCL2-associated agonist of cell death), which subsequently results in NFκB activation.

Although AKT3 and PRKCQ are involved in pro-survival pathways, their interaction is not fully understood. However, their interactions with other related protein kinases may offer biological clues on the mechanism by which AKT3 and PRKCQ synergistically influence aggressive PCA. For example, PRKCQ interacts with another AKT family member, AKT1, to activate NFκB [50]. If the AKT3-PRKCQ axis has a similar function as other protein kinases, namely AKT1 and PRKCQ, then these pro-survival markers may synergistically activate NFκB. As a result, activated NFκB may enable the tumor to escape programmed cell death and progress toward an aggressive PCA phenotype.

Numerous observational studies evaluated the impact of one or more apoptosis-related SNPs in relation to cancer outcomes [20-37]. Among 15 case-control studies, less than 10% of the sequence variants evaluated in the current study were significantly associated with various tumors, including of the colon, rectal, ovarian, breast, pancreatic, and non-small cell lung cancers [20-37]. In particular, modest cancer risk estimates were observed among 8 apoptosis-related SNPs detected in *CASP3, CASP8, CASP9, TP53, NFKB2*, and *NFKBIA*. However, some of these studies were limited by a small sample size, small number of analyzed SNPs, or failure to consider the impact of multiple SNPs on disease susceptibility. In the current study, there were 24 SNPs detected in 10 apoptosis-related genes [i.e., *AKT3, BIK, BNIP3L, CARD8, CASP9, IKBKE, PRKCE, TNFSF10*, and *TNFRSF10 *(*B, D*)]. These apoptosis-related SNPs, after adjusting for confounders, were modestly associated with PCA risk and/or aggressive disease. Yet, these findings lost statistical significance after adjusting for multiple hypothesis testing. Main effects were not observed for *AKT3 rs2125230 *and *PRKCQ rs571715 *in relation to PCA risk or disease progression in our study set. Wang and co-workers (2009) evaluated interactions among 5 apoptosis-related SNPs, including death receptor 4 (DR4), and pack-years of smoking in relation to bladder cancer using entropy-based MDR [51]. MDR analysis revealed a significant additive interaction between DR4 -397 G > T and smoking on bladder cancer. Unfortunately, this study analyzed a relatively small number of sequence variants in the DR4 apoptosis-related gene; hence, making it difficult to compare study findings. In a post-hoc analysis, we did not observe any interactions among the selected apoptosis-related markers and sources of reactive oxygen species, antioxidants, and anti-inflammatory agents, including cigarette smoking, dietary supplements, aspirin, ibuprofen, and meat-derived carcinogens (data not shown).

We considered the strengths, limitations and future directions of the current study. Although SEN-guided MDR only identified a nominally significant network for disease aggressiveness in the current study, this approach overcomes the computational challenge of detecting all possible two-, three- and four-way SNP combinations involved in PCA progression. SEN, in the current study, was used to prioritize > 10 million possible interactions by focusing on a "sub-network" of informative SNPs in relation to aggressive PCA. Reduction of our genetic data set to the most informative markers improved the feasibility to detect interactions that may have otherwise remained undetected. Unfortunately, the genome-wide association studies (GWAS) database used for the current study did not include apoptosis-related sequence variants (e.g., *TNF-308 rs1800629*, TNFSF10 *rs1131532, BCL2 -936 rs2279115*) previously reported in published cancer epidemiology studies [52-54]. Future studies in our laboratory will focus on high-throughput targeted sequencing to evaluate the impact of novel and commonly reported sequence variants on PCA susceptibility and disease prognosis. In light of recent GWAS reports, it is tempting to assume that extremely large case-control study sets, involving tens of thousands subjects are required to evaluate millions of SNP interactions in relation to PCA outcomes. However, the current study had adequate statistical power to evaluate individual and SNP combination effects in relation to prostate cancer. In particular, MDR has 80% statistical power to evaluate all possible two-, three-, and four-way gene-gene interactions with as low as 200 cases and 200 controls [55]. MDR remains effective even in the presence of 5% genotyping errors and/or 5% missing data [55]. It is anticipated that 5% of the about 13,000 possible interactions among 24 apoptosis-related SNPs will result in approximately 650 significant relationships due to chance alone. However, MDR coupled with permutation testing adjust for multiple comparison bias. Given the low prediction accuracy affiliated with the interaction between AKT3 and PRKCQ, our study findings require replication within independent study sets. However, recent simulation studies demonstrate that even modest disparities in genotype allele frequencies among study participants of independent study sets may interfere with the capacity to replicate complex interactions [56]. Thus, to ensure reproducibility within future studies, we plan to select study sets with similar genetic architecture (i.e., ancestry identification markers and SNP genotypes) as CGEMS PCA case-control subjects.

## Conclusion

In summary, we have identified a marginal interaction between two apoptosis-related SNPs linked to PCA disease aggressiveness using SEN-guided MDR. Future molecular and pre-clinical studies may help to clarify the functionality of these genetic variants and their role in PCA disease progression. Our state-of-the art bioinformatics approach will enable researchers to pre-process millions of SNP interactions using GWAS or cancer consortia genotype data. In order to replicate findings within independent study sets, researchers will have to ensure that comparative sub-populations have the same genetic architecture and ancestry backgrounds. Therefore, the application of SEN-guided MDR may ultimately help researchers identify and validate gene-gene and/or gene-environment interactions to serve as effective cancer prognostication tools.

## Methods

### Study population

The Prostate, Lung, Colon, and Ovarian (PLCO) Cancer Screening Trial is a randomized, well-designed, and multi-center investigation sponsored and coordinated by the National Cancer Institute (NCI) [57,58]. Between 1993 and 2001, the PLCO Trial recruited study participants aged 55 to 81 years old to evaluate the effect of screening on disease specific mortality, relative to standard care. Men randomized to the PCA arm of the trial, received annual PSA and DRE exams. For the current study, 2,286 European-American men were included in our study if they had GWAS data available through the Cancer Genetic Markers of Susceptibility (CGEMS) data portal. (http://cgems.cancer.gov/) [38]. The CGEMS study population consists of nationally available genetic data for nearly 500,000 sequence variants. Incident PCA cases (488 non-aggressive and 687 aggressive) were identified through various sources including: screening exams; reports from patients, physicians, or relatives; or linkage with the National Death Index or state cancer registries. Incident PCA cases were pathologically confirmed with aggressive (Gleason score ≥ 7 and tumor stage III/IV) or non-aggressive [Gleason score (< 7) and tumor stage I/II] disease. Controls (n = 1,111) were matched to cases identified based on age, time since initial screening, and year of blood draw using incidence density sampling. All participants signed informed consent documents approved by both the NCI and local institutional review boards. Access to clinical and background data collected through examinations and questionnaires was approved for use by the PLCO.

### Gene selection

A panel of 73 apoptosis-associated genes was selected from published cancer epidemiology or molecular biology studies as well as pathway databases and tools, including *Kyoto Encyclopedia of Genes and Genomes *(KEGG, http://www.genome.jp/kegg), Gene Ontology (GO), BioCarta (http://www.biocarta.com), ProteinLounge (http://www.proteinlounge.com), and Ingenuity (http://www.ingenuity.com) [59-63]. We searched PubMed for articles using the following keywords: [(prostate OR prostatic) AND (cancer OR neoplasms) AND apoptosis (gene variants OR single nucleotide polymorphisms OR targets) AND epidemiology]. Pathway tools, such as KEGG, BioCarta, ProteinLounge, and Ingenuity were used to visualize gene-gene and protein-protein interactions essential to regulating apoptosis [59-61,63]. The Entrez Gene database of the National Center for Biotechnology Information (*NCBI*, http://www.ncbi.nlm.nih.gov) was used to ensure that selected targets were involved in the apoptotic pathway [64].

### Criteria for SNP selection and data management

Prior to uploading our initial list (i.e., 73 apoptosis genes) into the CGEMS data portal, we secured the HUGO gene name equivalents for the targets of interest using NCBI Entrez Gene. SNP profile data was available for more than 1197 SNPs [38]. However, upon further investigation, many of these SNPs were eliminated if they were: not located within 2.5 kb of the 5' or 3' end of the gene; or detected within a gene that was not related to apoptosis. Among SNPs that were associated with apoptosis, we focused on gene regions located in coding, promoter, or "near gene" regions. "Near gene" regions were defined as 2.5 kb up- and downstream of the 5' or 3' ends of the selected genes, respectively.

We excluded any sequence variants that had a minor allele frequency (MAF) < 5% as reported in the NCBI Entrez SNP [64]. In addition, one SNP was removed since its genotype frequency distribution among controls deviated substantially from the Hardy-Weinberg Equilibrium, *p *≤ 0.005 (n = 1). Following data cleanup, 172 apoptotic SNPs were analyzed among 2,286 men of European descent (687 aggressive cases, 488 non-aggressive cases, and 1,111 controls).

### Predicted function of selected SNPs

The SNPinfo (http://snpinfo.niehs.nih.gov/) webserver enabled us to annotate and/or predict the functional consequence of selected apoptosis variants, as summarized in Additional file 2[65]. This server consists of several pipelines to predict whether alternative alleles of a SNP may alter one or more of the following: transcriptional regulation via transcription factor binding site (TFBS) activity; premature termination of amino-acid sequence (stop codons); the splicing pattern or efficiency at mRNA splice sites, exonic splicing enhancers (ESE) or silencers (ESS); protein structures or properties by changing single amino acids (i.e., non-synonymous SNPs); or mRNA transcription or protein translation by altering microRNA (miRNA) binding sites.

### Statistical analysis for single gene effects

Univariate and multivariate analyses were used to evaluate apoptosis associated SNPs among men of European descent in relation to PCA outcomes. To assess whether possession of one or more apoptotic alleles influence the risk of developing PCA, we tested for significant differences in the distribution of homozygous major, heterozygous, or homozygous minor genotypes between cases and controls using the chi-square test of heterogeneity. A case-only analysis was used to examine the relationship between apoptosis-related alleles and aggressive PCA. We evaluated differences in the distribution and inheritance of apoptosis-related genes comparing men with aggressive disease (Gleason score ≥ 7 and tumor stage > 2) to those with non-aggressive disease (i.e., other PCA cases that did not have both Gleason score ≥ 7 and tumor stage > 2). The associations between PCA outcomes and selected polymorphic genes, expressed as odds ratios (ORs) and corresponding 95% confidence intervals (CIs), were estimated using unconditional multivariate logistic regression (LR) models adjusted for age and family history of PCA. LR analyses for genetic variants and PCA outcomes were conducted using the major or common genotype as the referent category. All chi-square test and LR analyses were conducted using SAS 9.2 (SAS Institute Inc., Cary, NC) and SVS software (Golden Helix, Inc., Bozeman, MT). Statistical significance was assessed using a p-value < 0.05. Adjustments for multiple comparisons were made using Bonferroni correction and permutation testing.

### Statistical power

We performed calculations to determine the statistical power of our sample to detect significant relationships between apoptosis-related polymorphisms and PCA development. The expected risk estimates of our study were determined by specifying values for a number of parameters, including: an average minor allele frequency of at least 26.0%, NCI's estimate of PCA disease prevalence (19%); statistical power (80%); sample size (1,175 PCA cases and 1,111 disease-free individuals or 687 aggressive PCA cases and 488 non-aggressive cases). We assumed the outcome was in complete linkage disequilibrium with an apoptosis-predisposing variant (r^2 ^= 1.0). Based on the aforementioned parameters, we have > 80% power to detect genetic markers with odds ratios (ORs) of ≥ 1.4 (or 0.71 for protective effects) for PCA risk and ≥ 1.6 (or 0.62 for protective effects) for disease aggressiveness, assuming a codominant model with 1 degree of freedom (df). Power calculations were performed using Power for Genetic Association Version 2 Software [66].

### Analysis of gene interactions using multi-factor dimensionality reduction (MDR)

To evaluate the single- and joint- modifying effects of 172 candidate apoptosis-related SNPs within a large dataset, such as CGEMS, is computationally challenging [67,68]. In order to overcome this problem, open-source and freely available MDR 2.0 software (htpp://http://www.epistasis.org) was used to analyze interactions among apoptotic sequence variants in relation to PCA outcomes [69]. MDR is a data reduction approach, designed to detect and characterize high-order interactions in case-control studies. With MDR, information from various genetic loci is categorized into high-risk or low-risk groups of disease. The resulting one-dimensional multi-locus genotype variable is then evaluated for its ability to classify and predict a disease outcome through cross-validation and permutation testing. MDR uses a 10-fold cross validation to estimate the testing accuracy of a model by leaving out 1/10^th ^of the data as an independent test set. The model is developed on 9/10^th ^of the data and then evaluated on the remaining test set. This process is repeated for each 1/10^th ^of the data and the resulting prediction accuracies are averaged.

In the current study, the model with the greatest cross validation consistency (i.e., CVC ≥ 8/10) and highest prediction accuracy [i.e., Average Testing Accuracy (ATA)] was selected as the best predictor of disease outcome. Accuracy is a function of the percentage of true positives (TP), true negatives (TN), false positives (FP), and false negatives (FN) as defined as (TP + TN)/(TP + TN + FP + FN). ATAs are averaged across all 10 pieces of the data, in order to provide an estimate of the predictive ability of the loci in relation to the outcome of interest. We used cross-validation consistency (CVC) to measure the degree to which the same best MDR model was selected across the 10 divisions of the data. Models with a CVC of ≥ 8/10 using a 10-fold cross-validation were considered more carefully. If the MDR model met the CVC criteria, we selected models that had the highest ATAs. The combination of CVC and permutation testing were used to control for multiple hypothesis testing. Permutation testing results were statistically significant at the ≤ 0.05 level.

The current version of MDR used in this study enabled the incorporation and adjustment of multiple covariates [70]. To remove the covariate effects [i.e., age-group and family history of PCA (yes or no)], we integrated two sampling methods (i.e., over- and under-sampling). This approach is computationally efficient, since it allows for the adjustment of multiple covariates without significantly increasing computational burden.

### Visualization of interaction models using interaction entropy algorithms, hierarchical graphs and statistical epistasis network (SEN)

The interaction entropy algorithm, based on information theory, is a method to verify, visualize, and interpret combination effects identified by parametric (e.g., LR) and non-parametric (e.g., MDR) statistical test[43,71-74]. Jakulin and Brakto (2003) have developed a metric to gauge whether the gain in information (i.e., information gain) about a class variable (i.e., ability to predict disease status) from a combination of two variables provides more information than each variable considered independently [72,73]. In the current study, measures of interaction were used to build interaction entropy graphs to visualize and interpret interactions among selected apoptosis-related markers in relation to PCA risk and disease progression. Individual and all possible pairwise loci were assigned a mutual information (I) percentage score in relation to disease risk or aggressiveness; whereby typical scores for genetic loci are < 5%. Pairwise SNP combinations were deemed important if the joint mutual information [I = (SNP_1_, SNP_2_; disease status)] was greater than the total mutual information of each individual locus considered separately [I (SNP_1_; disease status) + I (SNP_2_; disease status)]. Interactions were further visualized using an interaction entropy graph, which uses a color-coding scheme to interpret interactions. Strong interacting factors, coded either red or orange, indicated high and medium levels of synergistic effects on outcomes, respectively. Weak interacting factors, coded either blue or green, denoted high or modest levels of redundancy between markers, respectively. Gold depicted independence and a midway point between synergy and redundancy. Design of interaction entropy graphs was accomplished using the Orange software [75].

An important limitation of any gene-gene interaction analysis method is the combinatorial nature of the problem. Exhaustive analysis of all possible two-, three-, four-way combinations of SNPs creates a computational burden and significant multiple testing problems. We addressed this issue by pre-processing the data using statistical epistasis network (SEN) modeling, which has been described elsewhere [39]. Briefly, this tool uses information theory, as previously described, to develop a network based on the amount of mutual information from SNP_1_, SNP_2_, and a class variable (i.e., disease status). Thus, it describes the pairwise interactive effect on a discrete outcome. An epistasis network, or graph, consists of a set of vertices (individual SNPs) and edges (pairwise interactions) that attach the vertices. In the current study, the statistical epistasis networks (SEN) were built by incrementally adding edges if their strengths were greater than a given threshold. Each vertex and each edge connecting two vertices were assigned a weight commensurate with the strength of main and interactive effects, respectively. The weight of a vertex is depicted by its size; whereby, a larger size is indicative of a higher main effect. Similarly, the thickness of the line between SNP pairs is directly proportional to the strength of their interaction.

We used SEN to visually summarize the strongest individual SNP effects and SNP-pairwise interactions. The topology of the graph, consisting of SNPs and their pairwise interactions, was used to guide MDR modeling. This resulted in a reduction in the total number of SNP combinations. For instance, we reduced the gene-gene interaction analysis from 172 to 24 SNPs that resided within the major connected component with the largest number of vertices (i.e., main effects). The derived major component plot was validated by comparing: (1) the number of vertices and edges among the various connected "sub-networks"; and (2) the size of the major component plot of real data versus randomized data that underwent 1000 permutations. The latter compared data using a pairwise interaction strength cut-off of 1% and significance level of 0.05. To ensure that no significant interactions were identified, non-informative SNPs outside the major component plot were also analyzed by MDR. In essence, SEN-guided MDR facilitated logical prioritization of SNPs for gene-gene interaction analysis in relation to PCA outcomes.

## Abbreviations

PCA: (PCA); SNP: (single nucleotide polymorphism); MDR: (multifactor dimensionality reduction); SEN: (Statistical Epistasis Network).

## Competing interests

The authors declare that they have no competing interests.

## Authors' contributions

NAL: conceptualized the project, conducted the statistical analysis and initial MDR modeling, interpreted study results, and composed the initial manuscript draft. ENR, SY, LRK: Intellectually contributed toward the introduction and/or discussion. TH: performed statistical epistasis network modeling. JZ and JR: conducted MDR modeling and/or SEN-guided MDR analysis. GNB & LRK: co-supervised data-management and statistical/bioinformatics analyses. LRK, DWH, GNB, JHM, KSK: served as mentors in the study design and implementation as well as data interpretation. All authors contributed to draft reviewing, editing, and approving the final manuscript draft.

## Pre-publication history

The pre-publication history for this paper can be accessed here:

http://www.biomedcentral.com/1755-8794/5/11/prepub

## Supplementary Material

Additional file 1 **Table S1**. Corresponding Gene and dbSNP IDs for apoptosis-related sequence variants depicted in the Statistical Epistasis Network Modeling Graph, presented in Figure 1. Click here for file

Additional file 2 **Table S2**. Functional consequence of selected apoptosis-related Polymorphisms. Click here for file

## References

[B1] American Cancer S Cancer Facts and Figures 2012 2012 Atlanta, Georgia: American Cancer Society

[B2] Tapia-Vieyra JV Mas-Oliva J Apoptosis and cell death channels in prostate cancer ArchMedRes 2001 32 3 175 185 10.1016/s0188-4409(01)00274-0 11395181

[B3] Zhivotovsky B Orrenius S Carcinogenesis and apoptosis: paradigms and paradoxes Carcinogenesis 2006 27 10 1939 1945 16606631 10.1093/carcin/bgl035

[B4] Limoli CL Hartmann A Shephard L Yang CR Boothman DA Bartholomew J Morgan WF Apoptosis, reproductive failure, and oxidative stress in Chinese hamster ovary cells with compromised genomic integrity Cancer Res 1998 58 16 3712 3718 9721883

[B5] Danial NN Korsmeyer SJ Cell death: critical control points Cell 2004 116 2 205 219 14744432 10.1016/s0092-8674(04)00046-7

[B6] Furuya Y Krajewski S Epstein JI Reed JC Isaacs JT Expression of bcl-2 and the progression of human and rodent prostatic cancers Clinical cancer research: an official journal of the American Association for Cancer Research 1996 2 2 389 398 9816182

[B7] Colombel M Symmans F Gil S O'Toole KM Chopin D Benson M Olsson CA Korsmeyer S Buttyan R Detection of the apoptosis-suppressing oncoprotein bc1-2 in hormone-refractory human prostate cancers Am J Pathol 1993 143 2 390 400 7688182 PMC1887010

[B8] McDonnell TJ Troncoso P Brisbay SM Logothetis C Chung LW Hsieh JT Tu SM Campbell ML Expression of the protooncogene bcl-2 in the prostate and its association with emergence of androgen-independent prostate cancer Cancer Res 1992 52 24 6940 6944 1458483

[B9] Fleischmann A Huland H Mirlacher M Wilczak W Simon R Erbersdobler A Sauter G Schlomm T Prognostic relevance of Bcl-2 overexpression in surgically treated prostate cancer is not caused by increased copy number or translocation of the gene The Prostate 2011 in press doi: 10.1002/pros.21504 10.1002/pros.21504 22024950

[B10] Abate-Shen C Shen MM Molecular genetics of prostate cancer Genes Dev 2000 14 19 2410 2434 11018010 10.1101/gad.819500

[B11] Thomas DJ Robinson M King P Hasan T Charlton R Martin J Carr TW Neal DE p53 expression and clinical outcome in prostate cancer Br J Urol 1993 72 5 Pt 2 778 781 8281412 10.1111/j.1464-410x.1993.tb16267.x

[B12] Shurbaji MS Kalbfleisch JH Thurmond TS Immunohistochemical detection of p53 protein as a prognostic indicator in prostate cancer Hum Pathol 1995 26 1 106 109 7821906 10.1016/0046-8177(95)90122-1

[B13] Stackhouse GB Sesterhenn IA Bauer JJ Mostofi FK Connelly RR Srivastava SK Moul JW p53 and bcl-2 immunohistochemistry in pretreatment prostate needle biopsies to predict recurrence of prostate cancer after radical prostatectomy J Urol 1999 162 6 2040 2045 10569564 10.1016/S0022-5347(05)68095-0

[B14] Pickard MR Edwards SE Cooper CS Williams GT Apoptosis regulators Fau and Bcl-G are down-regulated in prostate cancer Prostate 2010 70 14 1513 1523 20687224 10.1002/pros.21186

[B15] Boehm JS Zhao JJ Yao J Kim SY Firestein R Dunn IF Sjostrom SK Garraway LA Weremowicz S Richardson AL Integrative genomic approaches identify IKBKE as a breast cancer oncogene Cell 2007 129 6 1065 1079 17574021 10.1016/j.cell.2007.03.052

[B16] Guo JP Shu SK He L Lee YC Kruk PA Grenman S Nicosia SV Mor G Schell MJ Coppola D Deregulation of IKBKE is associated with tumor progression, poor prognosis, and cisplatin resistance in ovarian cancer Am J Pathol 2009 175 1 324 333 19497997 10.2353/ajpath.2009.080767 PMC2708818

[B17] Hill KM Kalifa S Das JR Bhatti T Gay M Williams D Taliferro-Smith L De Marzo AM The role of PI 3-kinase p110beta in AKT signally, cell survival, and proliferation in human prostate cancer cells Prostate 2010 70 7 755 764 20058239 10.1002/pros.21108

[B18] Gasparian AV Yao YJ Kowalczyk D Lyakh LA Karseladze A Slaga TJ Budunova IV The role of IKK in constitutive activation of NF-kappaB transcription factor in prostate carcinoma cells J Cell Sci 2002 115 Pt 1 141 151 11801732 10.1242/jcs.115.1.141

[B19] Jia S Liu Z Zhang S Liu P Zhang L Lee SH Zhang J Signoretti S Loda M Roberts TM Essential roles of PI(3)K-p110beta in cell growth, metabolism and tumorigenesis Nature 2008 454 7205 776 779 18594509 10.1038/nature07091 PMC2750091

[B20] Ricks-Santi L Mason T Apprey V Ahaghotu C McLauchlin A Josey D Bonney G Dunston GM p53 Pro72Arg polymorphism and prostate cancer in men of African descent Prostate 2010 70 16 1739 1745 20593380 10.1002/pros.21209 PMC3057117

[B21] Srivastava K Srivastava A Sharma KL Mittal B Candidate gene studies in gallbladder cancer: a systematic review and meta-analysis Mutat Res 2011 728 1-2 67 79 21708280 10.1016/j.mrrev.2011.06.002 PMC3162044

[B22] Lee EB Jeon HS Yoo SS Choi YY Kang HG Cho S Cha SI Choi JE Park TI Lee BH Polymorphisms in apoptosis-related genes and survival of patients with early-stage non-small-cell lung cancer Ann Surg Oncol 2010 17 10 2608 2618 20422457 10.1245/s10434-010-1082-4

[B23] Bye H Prescott NJ Matejcic M Rose E Lewis CM Parker MI Mathew CG Population-specific genetic associations with oesophageal squamous cell carcinoma in South Africa Carcinogenesis 2011 32 12 1855 1861 21926110 10.1093/carcin/bgr211 PMC3220606

[B24] Campa D Kaaks R Le Marchand L Haiman CA Travis RC Berg CD Buring JE Chanock SJ Diver WR Dostal L Interactions between genetic variants and breast cancer risk factors in the breast and prostate cancer cohort consortium J National Cancer Inst 2011 103 16 1252 1263 10.1093/jnci/djr265 PMC3156803 21791674

[B25] Broeks A Schmidt MK Sherman ME Couch FJ Hopper JL Dite GS Apicella C Smith LD Hammet F Southey MC Low penetrance breast cancer susceptibility loci are associated with specific breast tumor subtypes: findings from the Breast Cancer Association Consortium Hum Mol Genet 2011 20 16 3289 3303 21596841 10.1093/hmg/ddr228 PMC3140824

[B26] Couch FJ Wang X McWilliams RR Bamlet WR de Andrade M Petersen GM Association of breast cancer susceptibility variants with risk of pancreatic cancer Cancer epidemiology, biomarkers & prevention: a publication of the American Association for Cancer Research, cosponsored by the American Society of Preventive Oncology 2009 18 11 3044 3048 10.1158/1055-9965.EPI-09-0306 PMC3248348 19843670

[B27] Sergentanis TN Economopoulos KP Association of two CASP8 polymorphisms with breast cancer risk: a meta-analysis Breast Cancer Res Treat 2010 120 1 229 234 19629679 10.1007/s10549-009-0471-5

[B28] Enjuanes A Benavente Y Bosch F Martin-Guerrero I Colomer D Perez-Alvarez S Reina O Ardanaz MT Jares P Garcia-Orad A Genetic variants in apoptosis and immunoregulation-related genes are associated with risk of chronic lymphocytic leukemia Cancer Res 2008 68 24 10178 10186 19074885 10.1158/0008-5472.CAN-08-2221

[B29] Sigurdson AJ Bhatti P Doody MM Hauptmann M Bowen L Simon SL Weinstock RM Linet MS Rosenstein M Stovall M Polymorphisms in apoptosis- and proliferation-related genes, ionizing radiation exposure, and risk of breast cancer among U.S. Radiologic Technologists Cancer epidemiology, biomarkers & prevention: a publication of the American Association for Cancer Research, cosponsored by the American Society of Preventive Oncology 2007 16 10 2000 2007 10.1158/1055-9965.EPI-07-0282 17932347

[B30] Cox A Dunning AM Garcia-Closas M Balasubramanian S Reed MW Pooley KA Scollen S Baynes C Ponder BA Chanock S A common coding variant in CASP8 is associated with breast cancer risk Nat Genet 2007 39 3 352 358 17293864 10.1038/ng1981

[B31] Lan Q Zheng T Chanock S Zhang Y Shen M Wang SS Berndt SI Zahm SH Holford TR Leaderer B Genetic variants in caspase genes and susceptibility to non-Hodgkin lymphoma Carcinogenesis 2007 28 4 823 827 17071630 10.1093/carcin/bgl196

[B32] Lou Y Fang CQ Li JH A study on the expression of CASP9 gene and its polymorphism distribution in non-small cell lung cancer Zhonghua Yi Xue Yi Chuan Xue Za Zhi 2007 24 1 59 62 17285546

[B33] Consortium BCA Commonly studied single-nucleotide polymorphisms and breast cancer: results from the Breast Cancer Association Consortium J National Cancer Inst 2006 98 19 1382 1396 10.1093/jnci/djj374 17018785

[B34] Du J Huo J Shi J Yuan Z Zhang C Fu W Jiang H Yi Q Hou J Polymorphisms of nuclear factor-kappaB family genes are associated with development of multiple myeloma and treatment outcome in patients receiving bortezomib-based regimens Haematologica 2011 96 5 729 737 21228035 10.3324/haematol.2010.030577 PMC3084920

[B35] Gao J Pfeifer D He LJ Qiao F Zhang Z Arbman G Wang ZL Jia CR Carstensen J Sun XF Association of NFKBIA polymorphism with colorectal cancer risk and prognosis in Swedish and Chinese populations Scand J Gastroenterol 2007 42 3 345 350 17354114 10.1080/00365520600880856

[B36] Schildkraut JM Iversen ES Wilson MA Clyde MA Moorman PG Palmieri RT Whitaker R Bentley RC Marks JR Berchuck A Association between DNA damage response and repair genes and risk of invasive serous ovarian cancer PLoS One 2010 5 4 e10061 20386703 10.1371/journal.pone.0010061 PMC2851649

[B37] Sprague BL Trentham-Dietz A Garcia-Closas M Newcomb PA Titus-Ernstoff L Hampton JM Chanock SJ Haines JL Egan KM Genetic variation in TP53 and risk of breast cancer in a population-based case control study Carcinogenesis 2007 28 8 1680 1686 17449902 10.1093/carcin/bgm097

[B38] Yeager M Orr N Hayes RB Jacobs KB Kraft P Wacholder S Minichiello MJ Fearnhead P Yu K Chatterjee N Genome-wide association study of prostate cancer identifies a second risk locus at 8q24 Nat Genet 2007 39 5 645 649 17401363 10.1038/ng2022

[B39] Hu T Sinnott-Armstrong NA Kiralis JW Andrew AS Karagas MR Moore JH Characterizing genetic interactions in human disease association studies using statistical epistasis networks BMC Bioinforma 2011 12 364 10.1186/1471-2105-12-364 PMC3215301 21910885

[B40] Duell EJ Bracci PM Moore JH Burk RD Kelsey KT Holly EA Detecting pathway-based gene-gene and gene-environment interactions in pancreatic cancer Cancer epidemiology, biomarkers & prevention: a publication of the American Association for Cancer Research, cosponsored by the American Society of Preventive Oncology 2008 17 6 1470 1479 10.1158/1055-9965.EPI-07-2797 PMC4410856 18559563

[B41] Mason RA Morlock EV Karagas MR Kelsey KT Marsit CJ Schned AR Andrew AS EGFR pathway polymorphisms and bladder cancer susceptibility and prognosis Carcinogenesis 2009 30 7 1155 1160 19372140 10.1093/carcin/bgp077 PMC2704279

[B42] Andrew AS Karagas MR Nelson HH Guarrera S Polidoro S Gamberini S Sacerdote C Moore JH Kelsey KT Demidenko E DNA repair polymorphisms modify bladder cancer risk: a multi-factor analytic strategy HumHered 2008 65 2 105 118 10.1159/000108942 PMC2857629 17898541

[B43] Andrew AS Nelson HH Kelsey KT Moore JH Meng AC Casella DP Tosteson TD Schned AR Karagas MR Concordance of multiple analytical approaches demonstrates a complex relationship between DNA repair gene SNPs, smoking and bladder cancer susceptibility Carcinogenesis 2006 27 5 1030 1037 16311243 10.1093/carcin/bgi284

[B44] Lavender NA Benford ML VanCleave TT Brock GN Kittles RA Moore JH Hein DW Kidd LC Examination of polymorphic glutathione S-transferase (GST) genes, tobacco smoking and prostate cancer risk among men of African descent: a case-control study BMC Cancer 2009 9 397 19917083 10.1186/1471-2407-9-397 PMC2783040

[B45] Lavender NA Komolafe OO Benford M Brock G Moore JH Vancleave TT States JC Kittles RA Kidd LC No association between variant DNA repair genes and prostate cancer risk among men of African descent Prostate 2010 70 2 113 119 19760636 10.1002/pros.21048 PMC2798907

[B46] Chen M Kamat AM Huang M Grossman HB Dinney CP Lerner SP Wu X Gu J High-order interactions among genetic polymorphisms in nucleotide excision repair pathway genes and smoking in modulating bladder cancer risk Carcinogenesis 2007 28 10 2160 2165 17728339 10.1093/carcin/bgm167

[B47] Benford ML VanCleave TT Lavender NA Kittles RA Kidd LR 8q24 sequence variants in relation to prostate cancer risk among men of African descent: a case-control study BMC Cancer 2010 10 334 20584312 10.1186/1471-2407-10-334 PMC2912864

[B48] VanCleave TT Moore JH Benford ML Brock GN Kalbfleisch T Baumgartner RN Lillard JW Jr Kittles RA Kidd LC Interaction among variant vascular endothelial growth factor (VEGF) and its receptor in relation to prostate cancer risk Prostate 2010 70 4 341 352 19908237 10.1002/pros.21067 PMC4433472

[B49] Nakatani K Thompson DA Barthel A Sakaue H Liu W Weigel RJ Roth RA Up-regulation of Akt3 in estrogen receptor-deficient breast cancers and androgen-independent prostate cancer lines J Biol Chem 1999 274 31 21528 21532 10419456 10.1074/jbc.274.31.21528

[B50] Bauer B Krumbock N Fresser F Hochholdinger F Spitaler M Simm A Uberall F Schraven B Baier G Complex formation and cooperation of protein kinase C theta and Akt1/protein kinase B alpha in the NF-kappa B transactivation cascade in Jurkat T cells J Biol Chem 2001 276 34 31627 31634 11410591 10.1074/jbc.M103098200

[B51] Wang M Cheng G Zhang Z Fu G Genetic variants in the death receptor 4 gene contribute to susceptibility to bladder cancer Mutat Res 2009 661 1-2 85 92 19070628 10.1016/j.mrfmmm.2008.11.009

[B52] Nam RK Zhang WW Jewett MA Trachtenberg J Klotz LH Emami M Sugar L Sweet J Toi A Narod SA The use of genetic markers to determine risk for prostate cancer at prostate biopsy Clinical cancer research: an official journal of the American Association for Cancer Research 2005 11 23 8391 8397 16322300 10.1158/1078-0432.CCR-05-1226

[B53] Jung JH Chae YS Moon JH Kang BW Kim JG Sohn SK Park JY Lee MH Park HY TNF superfamily gene polymorphism as prognostic factor in early breast cancer J Cancer Res Clin Oncol 2010 136 5 685 694 19890662 10.1007/s00432-009-0707-0 PMC11827816

[B54] Kidd LR Coulibaly A Templeton TM Chen W Long LO Mason T Bonilla C Akereyeni F Freeman V Isaacs W Germline BCL-2 sequence variants and inherited predisposition to prostate cancer Prostate Cancer ProstaticDis 2006 9 3 284 292 10.1038/sj.pcan.4500884 16733517

[B55] Ritchie MD Hahn LW Moore JH Power of multifactor dimensionality reduction for detecting gene-gene interactions in the presence of genotyping error, missing data, phenocopy, and genetic heterogeneity Genetic epidemiology 2003 24 2 150 157 12548676 10.1002/gepi.10218

[B56] Greene CS Penrod NM Williams SM Moore JH Failure to replicate a genetic association may provide important clues about genetic architecture PLoS One 2009 4 6 e5639 19503614 10.1371/journal.pone.0005639 PMC2685469

[B57] Gohagan JK Prorok PC Hayes RB Kramer BS The Prostate, Lung, Colorectal and Ovarian (PLCO) Cancer Screening Trial of the National Cancer Institute: history, organization, and status Control ClinTrials 2000 21 6 Suppl 251S 272S 10.1016/s0197-2456(00)00097-0 11189683

[B58] Hayes RB Sigurdson A Moore L Peters U Huang WY Pinsky P Reding D Gelmann EP Rothman N Pfeiffer RM Methods for etiologic and early marker investigations in the PLCO trial MutatRes 2005 592 1-2 147 154 10.1016/j.mrfmmm.2005.06.013 16054167

[B59] Kanehisa M Araki M Goto S Hattori M Hirakawa M Itoh M Katayama T Kawashima S Okuda S Tokimatsu T KEGG for linking genomes to life and the environment Nucleic Acids Res 2008 36 Database D480 D484 18077471 10.1093/nar/gkm882 PMC2238879

[B60] Kanehisa M Goto S Hattori M oki-Kinoshita KF Itoh M Kawashima S Katayama T Araki M Hirakawa M From genomics to chemical genomics: new developments in KEGG Nucleic Acids Res 2006 34 Database D354 D357 16381885 10.1093/nar/gkj102 PMC1347464

[B61] Kanehisa M Goto S KEGG: kyoto encyclopedia of genes and genomes Nucleic Acids Res 2000 28 1 27 30 10592173 10.1093/nar/28.1.27 PMC102409

[B62] Ingenuity Systems 2008

[B63] Biocarta LLC 2000

[B64] National Center for Biotechnology Information 2011

[B65] Xu J Kibel AS Hu JJ Turner AR Pruett K Zheng SL Sun J Isaacs SD Wiley KE Kim ST Prostate cancer risk associated loci in African Americans Cancer EpidemiolBiomarkers Prev 2009 18 7 2145 2149 10.1158/1055-9965.EPI-09-0091 PMC2729762 19549807

[B66] Menashe I Rosenberg PS Chen BE PGA: power calculator for case-control genetic association analyses BMC Genet 2008 9 36 18477402 10.1186/1471-2156-9-36 PMC2387159

[B67] Greene CS Penrod NM Kiralis J Moore JH Spatially uniform relieff (SURF) for computationally-efficient filtering of gene-gene interactions BioData mining 2009 2 1 5 19772641 10.1186/1756-0381-2-5 PMC2761303

[B68] Moore JH Asselbergs FW Williams SM Bioinformatics challenges for genome-wide association studies Bioinformatics 2010 26 4 445 455 20053841 10.1093/bioinformatics/btp713 PMC2820680

[B69] Moore JH Gilbert JC Tsai CT Chiang FT Holden T Barney N White BC A flexible computational framework for detecting, characterizing, and interpreting statistical patterns of epistasis in genetic studies of human disease susceptibility J Theor Biol 2006 241 2 252 261 16457852 10.1016/j.jtbi.2005.11.036

[B70] Gui J Andrew AS Andrews P Nelson HM Kelsey KT Karagas MR Moore JH A simple and computationally efficient sampling approach to covariate adjustment for multifactor dimensionality reduction analysis of epistasis Hum Hered 2010 70 3 219 225 20924193 10.1159/000319175 PMC2982850

[B71] McGill WL Multivariate information transmission Psychometrika vol 1954 19 97 116

[B72] Jakulin A Bratko I Analyzing attribute interations Lecture Notes in Artificial Intelligence 2003 2838 229

[B73] Jakulin A Bratko I Smrke D Demsar J Supan B Attribute interactions in medical data analysis Lecture Notes in Artificial Intelligence 2003 2780 229

[B74] Moore JH Gilbert JC Tsai CT Chiang FT Holden T Barney N White BC A flexible computational framework for detecting, characterizing, and interpreting statistical patterns of epistasis in genetic studies of human disease susceptibility JTheorBiol 2006 241 2 252 261 10.1016/j.jtbi.2005.11.036 16457852

[B75] Mramor M Leban G Demsar J Zupan B Visualization-based cancer microarray data classification analysis Bioinformatics 2007 23 16 2147 2154 17586552 10.1093/bioinformatics/btm312

