# Supplementary material for: Interaction among apoptosis-associated sequence variants and joint effects on aggressive prostate cancer
Source: BMC Med Genomics. 2012 Apr 30;5:11. doi: 10.1186/1755-8794-5-11 (PMC3355002; doi:10.1186/1755-8794-5-11)
Supplement: Additional file 1 — Table S1. Corresponding Gene and dbSNP IDs for apoptosis-related sequence variants depicted in the Statistical Epistasis Network Modeling Graph, presented in Figure 1. [file 1755-8794-5-11-S1.DOCX]

| 0 | *ACIN1 rs1885097* | 46 | *BCL2L14 rs4763781* | 92 | *IKBKE rs944775* | 138 | *RIPK1 rs7739011* |
| --- | --- | --- | --- | --- | --- | --- | --- |
| 1 | *ACIN1 rs3751501* | 47 | *BCL2L14 rs4763782* | 93 | *JUN rs11688* | 139 | *RPS6KA1 rs11247963* |
| 2 | *AKT3 rs10157763* | 48 | *BCL2L14 rs879732* | 94 | *KRAS rs13096* | 140 | *RRAS rs1865077* |
| 3 | *AKT3 rs10803155* | 49 | *BCL2L14 rs885637* | 95 | *KRAS rs9266* | 141 | *TNFRSF10A rs1000294* |
| 4 | *AKT3 rs10927067* | 50 | *BCL2L14 rs885720* | 96 | *NFKB1 rs230547* | 142 | *TNFRSF10A rs13255394* |
| 5 | *AKT3 rs12031994* | 51 | *BCL2L14 rs888152* | 97 | *NFKB1 rs4648135* | 143 | *TNFRSF10A rs13278062* |
| 6 | *AKT3 rs2034915* | 52 | *BCL2L2 rs1950252* | 98 | *NFKB2 rs1056890* | 144 | *TNFRSF10A rs2230229* |
| 7 | *AKT3 rs2125230* | 53 | *BID rs181402* | 99 | *NFKBIA rs696* | 145 | *TNFRSF10A rs6557634* |
| 8 | *AKT3 rs2125231* | 54 | *BID rs181405* | 100 | *NFKBIA rs8904* | 146 | *TNFRSF10A rs7842021* |
| 9 | *AKT3 rs2345994* | 55 | *BID rs181408* | 101 | *NFKBIL1 rs2230365* | 147 | *TNFRSF10B rs1001793* |
| 10 | *AKT3 rs4132509* | 56 | *BID rs181417* | 102 | *PIK3CA rs1607237* | 148 | *TNFRSF10B rs1047266* |
| 11 | *AKT3 rs4614244* | 57 | *BID rs366542* | 103 | *PIK3CB rs500687* | 149 | *TNFRSF10B rs11135693* |
| 12 | *AKT3 rs897960* | 58 | *BID rs5746474* | 104 | *PIK3CG rs4727666* | 150 | *TNFRSF10B rs9644062* |
| 13 | *APAF1 rs10745834* | 59 | *BID rs5747351* | 105 | *PIK3CG rs4730205* | 151 | *TNFRSF10D rs1133782* |
| 14 | *APAF1 rs10860361* | 60 | *BID rs738095* | 106 | *PRKCA rs11656099* | 152 | *TNFRSF10D rs6651394* |
| 15 | *APAF1 rs1439123* | 61 | *BID rs9604787* | 107 | *PRKCA rs8074995* | 153 | *TNFRSF10D rs7463799* |
| 16 | *APAF1 rs1439124* | 62 | *BIK rs4988360* | 108 | *PRKCA rs9890506* | 154 | *TNFRSF10D rs7957* |
| 17 | *APAF1 rs2288714* | 63 | *BIK rs4988366* | 109 | *PRKCE rs1530668* | 155 | *TNFRSF1A rs1860545* |
| 18 | *APAF1 rs4319556* | 64 | *BNIP3L rs1042992* | 110 | *PRKCE rs17034455* | 156 | *TNFRSF1A rs4149570* |
| 19 | *APAF1 rs7299536* | 65 | *BNIP3L rs10503786* | 111 | *PRKCE rs2594489* | 157 | *TNFRSF1A rs4149576* |
| 20 | *APAF1 rs7315397* | 66 | *CARD8 rs10405717* | 112 | *PRKCE rs281472* | 158 | *TNFRSF1A rs4149577* |
| 21 | *APAF1 rs919699* | 67 | *CARD8 rs10416565* | 113 | *PRKCE rs281476* | 159 | *TNFRSF1A rs4149578* |
| 22 | *BAK1 rs210134* | 68 | *CARD8 rs11670259* | 114 | *PRKCE rs281505* | 160 | *TNFRSF1B rs1061622* |
| 23 | *BAK1 rs5745568* | 69 | *CARD8 rs11672725* | 115 | *PRKCE rs281508* | 161 | *TNFSF10 rs2270418* |
| 24 | *BAX rs11667351* | 70 | *CASP3 rs2019978* | 116 | *PRKCE rs3820729* | 162 | *TNFSF10 rs231983* |
| 25 | *BAX rs4645900* | 71 | *CASP6 rs3181187* | 117 | *PRKCE rs608139* | 163 | *TNFSF10 rs365238* |
| 26 | *BAX rs905238* | 72 | *CASP6 rs3212153* | 118 | *PRKCE rs935672* | 164 | *TNFSF10 rs4894559* |
| 27 | *BCL2 rs1016860* | 73 | *CASP6 rs768063* | 119 | *PRKCE rs935673* | 165 | *TNFSF10 rs9859259* |
| 28 | *BCL2 rs1564483* | 74 | *CASP7 rs12415607* | 120 | *PRKCE rs951012* | 166 | *TP53 rs2078486* |
| 29 | *BCL2 rs3927911* | 75 | *CASP8 rs10931934* | 121 | *PRKCQ rs2236379* | 167 | *TP53 rs2909430* |
| 30 | *BCL2A1 rs1138357* | 76 | *CASP8 rs6747918* | 122 | *PRKCQ rs2236380* | 168 | *TP53INP1 rs4735334* |
| 31 | *BCL2A1 rs1138358* | 77 | *CASP9 rs1052571* | 123 | *PRKCQ rs519951* | 169 | *TP53INP1 rs896849* |
| 32 | *BCL2A1 rs3826007* | 78 | *DFFA rs11588734* | 124 | *PRKCQ rs571715* | 170 | *TP53INP1 rs896854* |
| 33 | *BCL2L11 rs13405741* | 79 | *DFFB rs12738235* | 125 | *PRKCQ rs574521* | 171 | *TRAF2 rs3750512* |
| 34 | *BCL2L11 rs616130* | 80 | *DFFB rs3205087* | 126 | *PRKCQ rs585881* | 172 | *TRAF2 rs4880073* |
| 35 | *BCL2L11 rs724710* | 81 | *DFFB rs4074709* | 127 | *RAF1 rs11128607* |  |  |
| 36 | *BCL2L13 rs4488761* | 82 | *DFFB rs4648426* | 128 | *RAF1 rs11709504* |  |  |
| 37 | *BCL2L14 rs10772530* | 83 | *DIABLO rs12870* | 129 | *RAF1 rs11710163* |  |  |
| 38 | *BCL2L14 rs10845479* | 84 | *HRK rs7972948* | 130 | *RAF1 rs13060691* |  |  |
| 39 | *BCL2L14 rs11054704* | 85 | *HRK rs9669553* | 131 | *RAF1 rs6442322* |  |  |
| 40 | *BCL2L14 rs1612841* | 86 | *IKBIP rs1048906* | 132 | *RAF1 rs6792773* |  |  |
| 41 | *BCL2L14 rs1628766* | 87 | *IKBIP rs12371097* | 133 | *RAF1 rs7643321* |  |  |
| 42 | *BCL2L14 rs1641729* | 88 | *IKBIP rs12821083* | 134 | *RAF1 rs7956* |  |  |
| 43 | *BCL2L14 rs2448050* | 89 | *IKBKE rs11578093* | 135 | *RAF1 rs904453* |  |  |
| 44 | *BCL2L14 rs2448063* | 90 | *IKBKE rs1539243* | 136 | *RAF1 rs9817675* |  |  |
| 45 | *BCL2L14 rs4763780* | 91 | *IKBKE rs1930438* | 137 | *RELA rs7101916* |  |  |

Additional File 1. Corresponding Gene and dbSNP IDs for apoptosis-related sequence variants depicted in the statistical epistasis network modeling graph, presented in Figure 1.
