# Supplementary material for: Interaction among apoptosis-associated sequence variants and joint effects on aggressive prostate cancer
Source: BMC Med Genomics. 2012 Apr 30;5:11. doi: 10.1186/1755-8794-5-11 (PMC3355002; doi:10.1186/1755-8794-5-11)
Supplement: Additional file 2 — Table S2. Functional consequence of selected apoptosis-related Polymorphisms. [file 1755-8794-5-11-S2.DOC]

Additional File 2. Functional consequence of apoptosis-related polymorphisms

| dbSNP ID^†^ | Gene | Chr | Chr Position | Location | Nucleotide Change | Amino Acid Change | Predicted Functional Consequence |
| --- | --- | --- | --- | --- | --- | --- | --- |
| rs17126483 | *ACIN1* | 14 | 23561193 | Intron 2 | T >C |  | TFBS |
| rs1885097 | *ACIN1* | 14 | 22619159 | Exon 6 | A>G | Ser467Pro | Possibly Damaging |
| rs3751501 | *ACIN1* | 14 | 22619125 | Exon 2 | G>A | Ser478Phe | Possibly Damaging |
| rs10157763 | *AKT3* | 1 | 240321082 | Intron 1 | C>T |  |  |
| rs10803155 | *AKT3* | 1 | 240220823 | Intron 1 | G>A |  |  |
| rs10927067 | *AKT3* | 1 | 240249285 | Intron 1 | G>A |  |  |
| rs12031994 | *AKT3* | 1 | 240243350 | Intron 1 | T>C |  |  |
| rs2034915 | *AKT3* | 1 | 240204544 | Intron 1 | C>T |  |  |
| rs2125230 | *AKT3* | 1 | 240211889 | Intron 1 | G>A |  |  |
| rs2125231 | *AKT3* | 1 | 240088340 | Intron 1 | G>A |  |  |
| rs2345994 | *AKT3* | 1 | 240258316 | Intron 1 | C>T |  |  |
| rs4132509 | *AKT3* | 1 | 240269125 | Intron 1 | C>A |  |  |
| rs4614244 | *AKT3* | 1 | 240326915 | Intron 1 | T>C |  |  |
| rs897960 | *AKT3* | 1 | 240259609 | Intron 1 | A>G |  |  |
| rs10745834 | *APAF1* | 12 | 97562818 | Intron 1 | A>G |  |  |
| rs10860361 | *APAF1* | 12 | 97631965 | 3'near gene (-298) | A>G |  |  |
| rs1439123 | *APAF1* | 12 | 97578784 | Intron 1 | T>C |  |  |
| rs1439124 | *APAF1* | 12 | 97622538 | Intron 1 | A>C |  |  |
| rs2288714 | *APAF1* | 12 | 97610756 | Intron 1 | T>C |  |  |
| rs4319556 | *APAF1* | 12 | 97579054 | Intron 1 | G>A |  |  |
| rs7299536 | *APAF1* | 12 | 97603878 | Intron 1 | G>A |  |  |
| rs7315397 | *APAF1* | 12 | 97552834 | Intron 1 | G>A |  |  |
| rs919699 | *APAF1* | 12 | 97586794 | Intron 1 | C>T |  |  |
| rs210134 | *BAK1* | 6 | 33648187 | 3'near gene (+212) | G>A |  |  |
| rs5745568 | *BAK1* | 6 | 33656372 | 5'near gene (-265) | C>A |  | TFBS |
| rs11667351 | *BAX* | 19 | 54147966 | 5'near gene (-2477) | T>G |  | TFBS |
| rs1805419 | *BAX* | 19 | 49459104 | Intron 2 | G>A |  | TFBS |
| rs4645878 | *BAX* | 19 | 49457938 | 5'near gene (-248) | C>T |  | TFBS |
| rs4645900 | *BAX* | 19 | 54156175 | Intron 1 | C>T |  | TFBS, miRNA |
| rs905238 | *BAX* | 19 | 54157196 | 3'near gene (-85) | A>G |  | TFBS |
| rs1016860 | *BCL2* | 18 | 58946054 | 3'UTR (mRNA 1997) | G>A |  | miRNA |
| rs1564483 | *BCL2* | 18 | 58945634 | 3'UTR (mRNA 2417) | G>A |  | miRNA |
| rs3927911 | *BCL2* | 18 | 59084606 | Intron 1 | C>T |  |  |
| rs4987721 | *BCL2* | 18 | 60954063 | Intron 2 | A>G |  |  |
| rs1138357 | *BCL2A1* | 15 | 78050461 | Exon 1 | G>A | Cys19Tyr | Benign |
| rs1138358 | *BCL2A1* | 15 | 78050400 | Exon 1 | T>G | Asn39Lys | Benign |
| rs3826007 | *BCL2A1* | 15 | 78050272 | Exon 1 | G>A | Gly82Asp | Possibly Damaging |
| rs13405741 | *BCL2L11* | 2 | 111629287 | Intron 1 | T>C |  |  |
| rs3789068 | *BCL2L11* | 2 | 111909247 | Intron 5 | T>C |  |  |
| rs616130 | *BCL2L11* | 2 | 111628912 | Intron 1 | C>A |  |  |

| dbSNP ID^†^ | Gene | Chr | Chr Position | Location | Nucleotide Change | Amino Acid Change | Predicted Functional Consequence |
| --- | --- | --- | --- | --- | --- | --- | --- |
| rs724710 | *BCL2L11* | 2 | 111623922 | Exon 1 | C>T | Ile95Ile | Altered Splicing |
| rs10772530 | *BCL2L14* | 12 | 12157308 | Intron 1 | C>A |  |  |
| rs10845479 | *BCL2L14* | 12 | 12135568 | Intron 1 | A>G |  |  |
| rs11054704 | *BCL2L14* | 12 | 12191036 | Intron 1 | G>A |  |  |
| rs1612841 | *BCL2L14* | 12 | 12136155 | Intron 1 | T>C |  |  |
| rs1628766 | *BCL2L14* | 12 | 12142116 | Intron 1 | T>C |  |  |
| rs1641729 | *BCL2L14* | 12 | 12142419 | Intron 1 | G>A |  |  |
| rs2448050 | *BCL2L14* | 12 | 12140491 | Intron 1 | G>A |  |  |
| rs2448063 | *BCL2L14* | 12 | 12113301 | 5'near gene (-1844) | G>T |  | TFBS |
| rs4763782 | *BCL2L14* | 12 | 12146023 | Intron 1 | A>C |  |  |
| rs879732 | *BCL2L14* | 12 | 12131466 | Exon 1 | T>C | Tyr162Tyr | Splice Site |
| rs885637 | *BCL2L14* | 12 | 12139547 | Intron 1 | G>A |  |  |
| rs885720 | *BCL2L14* | 12 | 12139366 | Intron 1 | G>A |  |  |
| rs888152 | *BCL2L14* | 12 | 12131932 | Intron 1 | C>T |  |  |
| rs1950252 | *BCL2L2* | 14 | 22848538 | 3'UTR (mRNA 1294) | G>A |  | miRNA |
| rs181402 | *BID* | 22 | 16604328 | Intron 1 | C>T |  |  |
| rs181405 | *BID* | 22 | 16607554 | Intron 1 | G>A |  |  |
| rs181408 | *BID* | 22 | 16609859 | Intron 1 | T>C |  |  |
| rs181417 | *BID* | 22 | 16615528 | Intron 1 | G>A |  |  |
| rs366542 | *BID* | 22 | 16632936 | 5'near gene (+1137) | C>T |  | TFBS |
| rs5746474 | *BID* | 22 | 16620486 | Intron 1 | T>C |  |  |
| rs5747351 | *BID* | 22 | 16620929 | Intron 1 | A>G |  |  |
| rs738095 | *BID* | 22 | 16630542 | Intron 1 | G>A |  | TFBS |
| rs9604787 | *BID* | 22 | 16609143 | Intron 1 | G>A |  |  |
| rs4988360 | *BIK* | 22 | 41829569 | 5'near gene (-1725) | C>T |  | TFBS |
| rs4988366 | *BIK* | 22 | 41830193 | 5'near gene (-1101) | A>G |  | TFBS |
| rs1042992 | *BNIP3L* | 8 | 26325108 | 3'UTR (mRNA 2047) | C>T |  | miRNA |
| rs10503786 | *BNIP3L* | 8 | 26325853 | 3'UTR (mRNA 2792) | C>T |  | miRNA |
| rs10405717 | *CARD8* | 19 | 53443620 | Intron 1 | C>T |  |  |
| rs10416565 | *CARD8* | 19 | 53434199 | Intron 1 | A>G |  |  |
| rs11670259 | *CARD8* | 19 | 53435976 | Intron 1 | C>T |  |  |
| rs11672725 | *CARD8* | 19 | 53438493 | Intron 1 | C>T |  |  |
| rs6509364 | *CARD8* | 19 | 53433879 | Intron 2 | C>T |  |  |
| rs6509366 | *CARD8* | 19 | 53435389 | Intron 2 | G>A |  |  |
| rs6509364 | *CARD8* | 19 | 53433879 | Intron 2 | C>T |  |  |
| rs6509366 | *CARD8* | 19 | 53435389 | Intron 2 | G>A |  |  |

| dbSNP ID^†^ | Gene | Chr | Chr Position | Location | Nucleotide Change | Amino Acid Change | Predicted Functional Consequence |
| --- | --- | --- | --- | --- | --- | --- | --- |
| rs2019978 | *CASP3* | 4 | 185932730 | Intron 1 | T>G |  |  |
| rs3181187 | *CASP6* | 4 | 110980514 | Intron 1 | G>A |  |  |
| rs3212153 | *CASP6* | 4 | 110967085 | 3'near gene (+337) | C>T |  | miRNA |
| rs768063 | *CASP6* | 4 | 110978060 | Intron 1 | G>A |  |  |
| rs12415607 | *CASP7* | 10 | 115428194 | 5'near gene (-740) | C>A |  | TFBS |
| rs10931934 | *CASP8* | 2 | 201945295 | Intron | C>T |  |  |
| rs1052571 | *CASP9* | 1 | 15595919 | Exon 1 | C>T | Ala28Val | Splicing Site, Benign |
| rs1052576 | *CASP9* | 1 | 15832543 | Exon 5 | G>A | Arg221Gln | Splicing Site, Benign |
| rs11588734 | *DFFA* | 1 | 10467247 | 5'near gene (-419) | A>G |  | TFBS |
| rs3205087 | *DFFB* | 1 | 3823399 | Exon 1 | G>A | Pro318Pro |  |
| rs4074709 | *DFFB* | 1 | 3820105 | Intron 1 | G>T |  |  |
| rs4648426 | *DFFB* | 1 | 3796246 | 5'near gene (+756) | T>C |  | TFBS |
| rs12870 | *DIABLO* | 12 | 121217700 | 3'UTR (mRNA 1633) | G>A |  | miRNA |
| rs7972948 | *HRK* | 12 | 115774919 | Intron 1 | C>T |  |  |
| rs9669553 | *HRK* | 12 | 115778069 | Intron 1 | C>T |  |  |
| rs1048906 | *IKBIP* | 12 | 97531754 | Exon 1 | G>A | Gly265Ser | miRNA, Benign |
| rs12371097 | *IKBIP* | 12 | 97531767 | Intron 1 | A>G |  |  |
| rs12821083 | *IKBIP* | 12 | 97533519 | Intron 1 | G>T |  |  |
| rs11578093 | *IKBKE* | 1 | 203059049 | 3'near gene (+495) | T>G |  |  |
| rs1539243 | *IKBKE* | 1 | 203036182 | Exon 4 | C>T | Ile67Ile | Splice Site |
| rs1930438 | *IKBKE* | 1 | 203031501 | 5'near gene (-710) | G>A |  | TFBS |
| rs944775 | *IKBKE* | 1 | 203056988 | Intron 1 | A>G |  |  |
| rs11688 | *JUN* | 1 | 58960014 | Exon 1 | G>A | Gln250Gln |  |
| rs13096 | *KRAS* | 12 | 25251108 | 3'UTR (mRNA 3636) | A>G |  | miRNA |
| rs9266 | *KRAS* | 12 | 25253484 | 3'UTR (mRNA 1260) | T>C |  | miRNA |
| rs230547 | *NFKB1* | 4 | 103893462 | Intron 1 | C>T |  |  |
| rs9266 | *KRAS* | 12 | 25253484 | 3'UTR (mRNA 1260) | T>C |  | miRNA |
| rs230547 | *NFKB1* | 4 | 103893462 | Intron 1 | C>T |  |  |
| rs4648135 | *NFKB1* | 4 | 103893871 | Intron 1 | A>G |  |  |

| dbSNP ID^†^ | Gene | Chr | Chr Position | Location | Nucleotide Change | Amino Acid Change | Predicted Functional Consequence |
| --- | --- | --- | --- | --- | --- | --- | --- |
| rs1056890 | *NFKB2* | 10 | 104152760 | 3'near gene (-523) | C>T |  | miRNA |
| rs696 | *NFKBIA* | 14 | 34940844 | 3'UTR (mRNA 1190) | G>A |  | miRNA |
| rs2230365 | *NFKBIL1* | 6 | 31633427 | Exon 2 | C>T | Ser126Ser | Splice Site, miRNA |
| rs1607237 | *PIK3CA* | 3 | 180432999 | Intron 1 | T>C |  |  |
| rs500687 | *PIK3CB* | 3 | 139942901 | Intron 1 | T>C |  |  |
| rs4727666 | *PIK3CG* | 7 | 106099429 | 5'near gene (+473) | A>G |  | TFBS |
| rs4730205 | *PIK3CG* | 7 | 106123200 | Intron 1 | C>T |  |  |
| rs11656099 | *PRKCA* | 17 | 62224124 | Intron 1 | G>A |  |  |
| rs8074995 | *PRKCA* | 17 | 62222593 | Intron 1 | G>A |  |  |
| rs9890506 | *PRKCA* | 17 | 62214645 | Intron 1 | C>T |  | TFBS |
| rs1530668 | *PRKCE* | 2 | 46318270 | Intron 1 | T>C |  |  |
| rs17034455 | *PRKCE* | 2 | 46225132 | Exon 4 | C>T | His524His | Altered Splicing |
| rs2594489 | *PRKCE* | 2 | 46319722 | Intron 1 | C>T |  |  |
| rs281472 | *PRKCE* | 2 | 46315488 | Intron 1 | T>C |  |  |
| rs281476 | *PRKCE* | 2 | 46312255 | Intron 1 | T>C |  |  |
| rs281505 | *PRKCE* | 2 | 46427196 | 3'near gene (+417) | T>C |  |  |
| rs281508 | *PRKCE* | 2 | 46322858 | Intron 1 | G>T |  |  |
| rs3820729 | *PRKCE* | 2 | 46304426 | Intron 1 | G>A |  |  |
| rs608139 | *PRKCE* | 2 | 45789207 | 5'near gene (+1360) | T>C |  | TFBS |
| rs935672 | *PRKCE* | 2 | 45957610 | Intron 9 | C>T |  |  |
| rs935673 | *PRKCE* | 2 | 46034008 | Intron 9 | A>G |  |  |
| rs951012 | *PRKCE* | 2 | 46309282 | Intron 1 | C>A |  |  |
| rs2236379 | *PRKCQ* | 10 | 6567149 | Exon 3 | C>T | Pro330Leu | Splice Site, Benign |
| rs2236380 | *PRKCQ* | 10 | 6509823 | 3'UTR (mRNA 2458) | C>T |  | miRNA |
| rs519951 | *PRKCQ* | 10 | 6598346 | 5'near gene (-1243) | C>T |  |  |
| rs571715 | *PRKCQ* | 10 | 6596560 | Intron 1 | T>C |  |  |
| rs574521 | *PRKCQ* | 10 | 6596860 | Intron 1 | T>C |  |  |
| rs585881 | *PRKCQ* | 10 | 6598275 | 5'near gene (-1172) | T>C |  |  |
| rs11128607 | *RAF1* | 3 | 12648275 | Intron 1 | A>G |  |  |
| rs11709504 | *RAF1* | 3 | 12649199 | Intron 1 | T>C |  |  |
| rs11710163 | *RAF1* | 3 | 12671288 | Intron 1 | A>G |  |  |
| rs13060691 | *RAF1* | 3 | 12653013 | Intron 1 | T>G |  |  |
| rs6442322 | *RAF1* | 3 | 12664858 | Intron 1 | A>G |  |  |
| rs6792773 | *RAF1* | 3 | 12643726 | Intron 1 | C>T |  |  |
| rs7643321 | *RAF1* | 3 | 12638233 | Intron 1 | A>G |  |  |
| rs7956 | *RAF1* | 3 | 12599763 | 5'near gene (+468) | A>G |  | miRNA |
| rs904453 | *RAF1* | 3 | 12679894 | Intron 1 | C>A |  | TFBS |
| rs9817675 | *RAF1* | 3 | 12651113 | Intron 1 | C>T |  |  |
| rs7101916 | *RELA* | 11 | 65187936 | 5'near gene (-883) | C>T |  | TFBS |

| dbSNP ID^†^ | Gene | Chr | Chr Position | Location | Nucleotide Change | Amino Acid Change | Predicted Functional Consequence |
| --- | --- | --- | --- | --- | --- | --- | --- |
| rs7739011 | *RIPK1* | 6 | 3057529 | Intron 1 | T>C |  |  |
| rs11247963 | *RPS6KA1* | 1 | 26539543 | 5'near gene (+722) | G>A |  | TFBS |
| rs1865077 | *RRAS* | 19 | 54831904 | Exon 3 | T>C | Asn111Asn | Splice Site |
| rs1000294 | *TNFRSF10A* | 8 | 23136080 | Intron 1 | C>T |  |  |
| rs13255394 | *TNFRSF10A* | 8 | 23139491 | 5'near gene (-890) | C>T |  | TFBS |
| rs13278062 | *TNFRSF10A* | 8 | 23138916 | 5'near gene (-386) | G>T |  |  |
| rs2230229 | *TNFRSF10A* | 8 | 23105237 | Exon 7 | A>G | Arg441Lys | Benign |
| rs6557634 | *TNFRSF10A* | 8 | 23116201 | Exon 4 | T>C | His141Arg | Probably Damaging |
| rs7842021 | *TNFRSF10A* | 8 | 23135149 | Intron 1 | A>G |  |  |
| rs1001793 | *TNFRSF10B* | 8 | 22956894 | Intron 1 | G>A |  |  |
| rs1047266 | *TNFRSF10B* | 8 | 22956646 | Exon 2 | C>T | Ala67Val | Benign |
| rs11135693 | *TNFRSF10B* | 8 | 22981099 | Intron 1 | C>A |  |  |
| rs9644062 | *TNFRSF10B* | 8 | 22976094 | Intron 1 | C>T |  |  |
| rs1133782 | *TNFRSF10D* | 8 | 23057933 | Exon 6 | C>T | Ser310Leu | Benign |
| rs6651394 | *TNFRSF10D* | 8 | 23078565 | 5'near gene (-957) | T>C |  | TFBS |
| rs7463799 | *TNFRSF10D* | 8 | 23075599 | Intron 1 | T>C |  |  |
| rs7957 | *TNFRSF10D* | 8 | 23049312 | 3'UTR (mRNA 3269) | T>C |  | miRNA |
| rs1860545 | *TNFRSF1A* | 12 | 6317038 | Intron 1 | C>T |  |  |
| rs4149570 | *TNFRSF1A* | 12 | 6321851 | 5'near gene (+222) | G>T |  | TFBS |
| rs4149576 | *TNFRSF1A* | 12 | 6319376 | Intron 1 | A>G |  |  |
| rs4149577 | *TNFRSF1A* | 12 | 6317783 | Intron 1 | T>C |  |  |
| rs4149578 | *TNFRSF1A* | 12 | 6317698 | Intron 1 | G>A |  |  |
| rs1061622 | *TNFRSF1B* | 1 | 12187221 | Exon 4 | T>G | Met196Arg | Splice Site, Benign |
| rs2270418 | *TNFSF10* | 3 | 173723701 | Intron 1 | T>G |  | TFBS |
| rs231983 | *TNFSF10* | 3 | 173719142 | Intron 1 | A>C |  |  |
| rs365238 | *TNFSF10* | 3 | 173724568 | 5'near gene (-544) | A>G |  | TFBS |
| rs4894559 | *TNFSF10* | 3 | 173716071 | Intron 1 | G>A |  |  |
| rs9859259 | *TNFSF10* | 3 | 173705843 | 3'near gene (+356) | C>A |  |  |
| rs2078486 | *TP53* | 17 | 7523808 | Intron 1 | G>A |  |  |
| rs2909430 | *TP53* | 17 | 7519370 | 5'UTR (mRNA 167) | A>G |  |  |
| rs4735334 | *TP53INP1* | 8 | 96024468 | Intron 1 | A>G |  |  |
| rs896849 | *TP53INP1* | 8 | 96011420 | 3'UTR (mRNA 1570) | T>C |  | miRNA |
| rs896854 | *TP53INP1* | 8 | 96029687 | Intron 1 | A>G |  |  |
| rs3750512 | *TRAF2* | 9 | 137096905 | 3'near gene (-2) | T>C |  |  |
| rs4880073 | *TRAF2* | 9 | 137093190 | Intron 1 | G>A |  |  |

Abbreviations: Chr, chromosome; UTR, untranslated region; ^†^173 apoptosis-related SNPs were analyzed among European-American male participants of the CGEMS study.
